# Exercise-acclimated microbiota improves skeletal muscle metabolism via circulating bile acid deconjugation

**DOI:** 10.1016/j.isci.2023.106251

**Published:** 2023-02-21

**Authors:** Wataru Aoi, Ryo Inoue, Katsura Mizushima, Akira Honda, Marie Björnholm, Tomohisa Takagi, Yuji Naito

**Affiliations:** 1 Division of Applied Life Sciences, Graduate School of Life and Environmental Sciences, Kyoto Prefectural University, Kyoto 6068522, Japan; 2 Laboratory of Animal Science, Department of Applied Biological Sciences, Faculty of Agriculture, Setsunan University, Osaka 5730101, Japan; 3 Department of Human Immunology and Nutrition Science, Graduate School of Medical Science, Kyoto Prefectural University of Medicine, Kyoto 6028566, Japan; 4 Gastroenterology, Tokyo Medical University Ibaraki Medical Center, Ibaraki 3000395, Japan; 5 Department of Molecular Medicine and Surgery, Karolinska Institutet, Stockholm 17176, Sweden; 6 Molecular Gastroenterology and Hepatology, Graduate School of Medical Science, Kyoto Prefectural University of Medicine, Kyoto 6028566, Japan; 7 Department for Medical Innovation and Translational Medical Science, Graduate School of Medical Science, Kyoto Prefectural University of Medicine, Kyoto 6028566, Japan

**Keywords:** Musculoskeletal medicine, Microbiome, Microbial metabolism

## Abstract

Habitual exercise alters the intestinal microbiota composition, which may mediate its systemic benefits. We examined whether transplanting fecal microbiota from trained mice improved skeletal muscle metabolism in high-fat diet (HFD)-fed mice. Fecal samples from sedentary and exercise-trained mice were gavage-fed to germ-free mice. After receiving fecal samples from trained donor mice for 1 week, recipient mice had elevated levels of AMP-activated protein kinase (AMPK) and insulin growth factor-1 in skeletal muscle. In plasma, bile acid (BA) deconjugation was found to be promoted in recipients transplanted with feces from trained donor mice; free-form BAs also induced more AMPK signaling and glucose uptake than tauro-conjugated BAs. The transplantation of exercise-acclimated fecal microbiota improved glucose tolerance after 8 weeks of HFD administration. Intestinal microbiota may mediate exercise-induced metabolic improvements in mice by modifying circulating BAs. Our findings provide insights into the prevention and treatment of metabolic diseases.

## Highlights

• Habitual exercise modulates the composition of the gut microbiota
• The modulation of microbiota ameliorates diet-induced metabolic disturbance
• Deconjugated BAs activate AMPK signaling and glucose uptake in skeletal muscle
• Altered microbiota improves metabolism by modifying circulating bile acids in mice

## Graphical abstract

**Figure:**
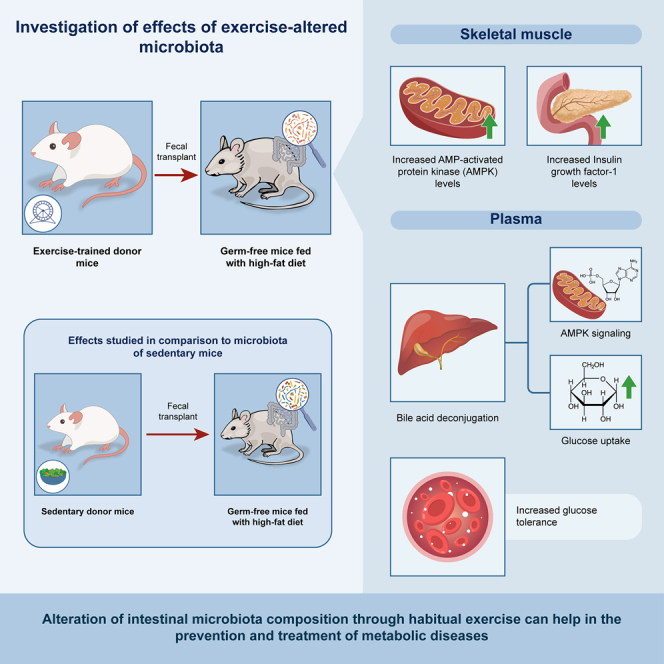

## Introduction

Metabolic dysfunction is involved in the pathogenesis of several non-communicable diseases, including type 2 diabetes, cardiovascular diseases, and cancer. Physical inactivity, along with overeating and an unbalanced diet, results in pre-conditions of non-communicable diseases such as hyperglycemia and dyslipidemia.^1^^,^^2^ By contrast, habitual exercise reduces the risk of non-communicable diseases^3^^,^^4^ through the improvement of metabolic states including insulin sensitivity, mitochondrial respiration, and protein synthesis.^5^ Skeletal muscle is an important metabolic and exercise-responsive organ, responsible for respiratory mechanics, maintaining posture and balance in addition to protecting vital organs. Regulation of protein synthesis via growth factors/mTOR pathway and glucose uptake via insulin receptor/Akt and the AMP-activated kinase (AMPK) pathways are well known as crucial exercise-inducible signal transduction processes in skeletal muscle. Hence, metabolic improvement of skeletal muscle maintains glucose, protein, and energy homeostasis in the entire body. Exercise-responsive mechanisms that enhance skeletal muscle metabolism are beneficial for the maintenance of systemic metabolic states.

Accumulating evidence shows that microbiota regulates intestinal conditions, systemic immune functions, and metabolic systems of the host. The gut microbiota is formed from bacteria colonizing the guts of animals and humans, consisting of approximately 300 trillion bacteria from more than 1,000 species. Dysbiosis of gut microbiota causes metabolic dysfunction in the host, including obesity, insulin resistance, and dyslipidemia.^6^^,^^7^ In reverse, daily physical activity modulates the microbiota profile whereby cross-sectional studies indicate that subjects who perform physical activity have more bacteria associated with metabolism and immune functions than subjects who led a sedentary lifestyle.^8^^,^^9^ Exercise intervention also beneficially changes the microbiota composition, suggesting immune and metabolic regulation in humans and animals.^9^^,^^10^ In addition, previous reports show that modulation of exercise-induced microbiota contributes to reduced pathogenic bacterial communities and increased bacteria that produce beneficial metabolites such as short-chain fatty acids, butyrate, and antioxidants.^11^ Hence, the microbiota composition may be closely involved in exercise-induced metabolic benefits. Nevertheless, the association between exercise-induced metabolic improvement and microbiota changes remains unknown.

Skeletal muscle metabolism is influenced by various hormones, cytokines, and nutrients. Moreover, metabolites secreted from the intestine into the circulation affect muscle metabolic functions as proven in leaky gut and dysbiotic conditions whereby typical gut-derived metabolites and endotoxins are regularly elevated, leading to low-grade inflammation and metabolic dysfunction.^12^^,^^13^^,^^14^ Given that exercise-induced microbiota change is more established in diet-induced obese mice and humans,^15^^,^^16^ this may prevent inflammation and metabolic dysfunction in skeletal muscle. Nevertheless, improvements in microbiota-derived muscle metabolism due to habitual exercise remain unclear. Here, we hypothesized that exercise-acclimated microbiota may ameliorate muscle metabolism by affecting circulating metabolites. To test this hypothesis, comprehensive information on blood metabolites and skeletal muscle mRNA expression in recipients was essential, which in turn enabled our discovery on the unique mechanisms and microbiota functions that support the communication between muscle and gut. We further postulate that our findings may guide the development of potential strategies for the prevention and treatment of metabolic diseases. Herein, we examined the effect of transplanting fecal microbiota from exercise-trained mice into diet-induced obese mice, on the circulating metabolite profile and skeletal muscle metabolism.

## Results

### Microbiota modulation in donor and recipient mice by habitual exercise

**Figure 1:**
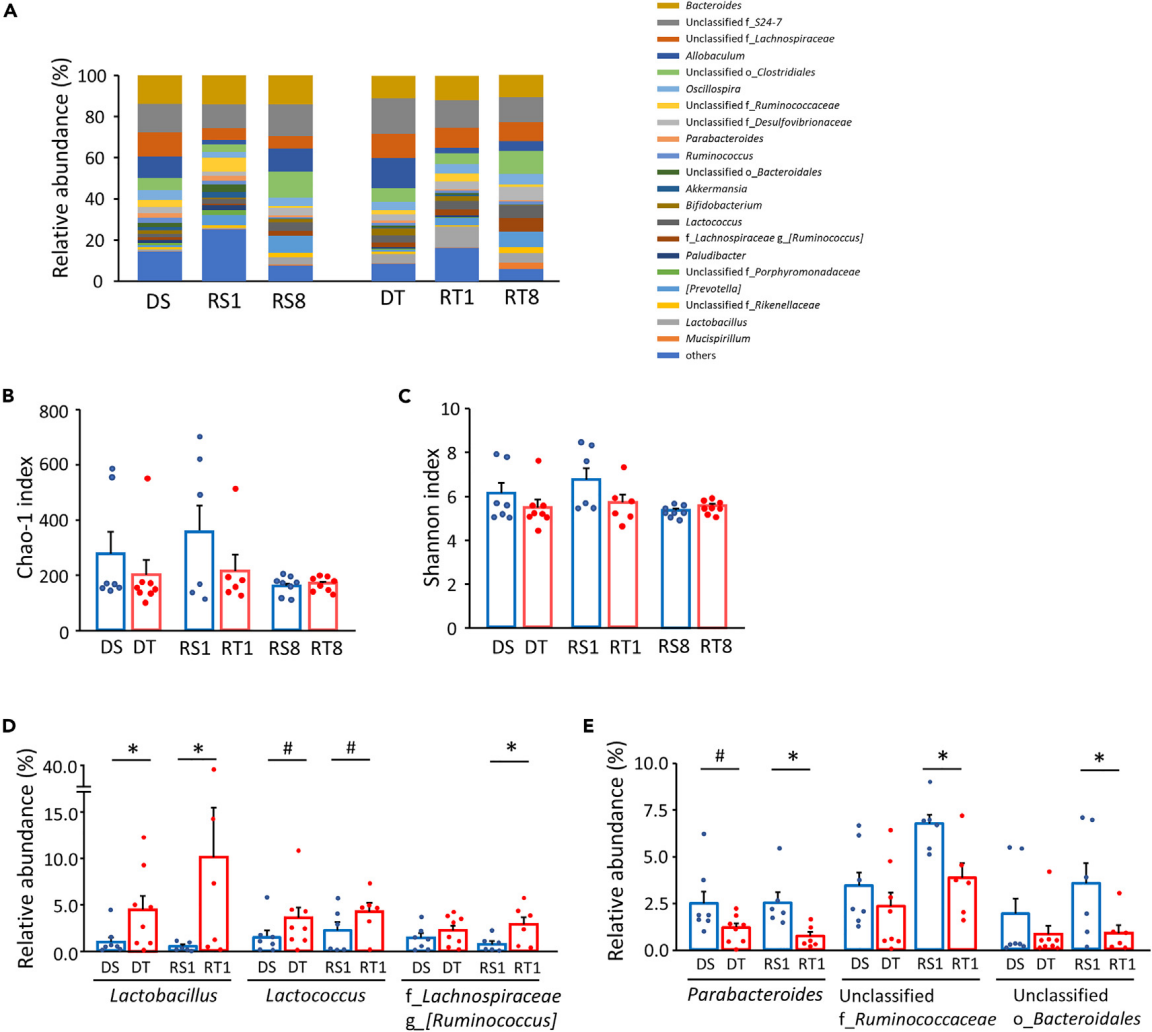
Microbiota profile in donor and recipient mice The relative abundance of genera in fecal microbiota profiles from donor and at weeks 1 and 8 of recipient mice (abundant >2% amplicon sequence variant [ASV] in at least one group) (n = 6–8, A). The α-diversity (Chao1 index [n = 6–8, B], ASV richness estimation and the Shannon index [n = 6–8, C], ASV evenness estimation) between the donor and recipient groups. The relative abundance of the genera that were increased (D) and decreased (E) by training in donor and at week 1 of recipient mice (n = 6–8). DS; sedentary donor, DT; trained donor, RS1; week 1-recipient from DS, RT1; week 1-recipient from DT, RS8; week 8-recipient from DS, RT8; week 8-recipient from DT. ^#^p < 0.1 and ∗p < 0.05. Results are presented as means ± SE.

In germ-free mice, the first exposure to microbiota dominantly affects its composition.^17^ Thereafter, continuous exposure further establishes the composition even in conventional housing, as described in a previous study.^18^ Thus, we examined the microbiota profile obtained from donor-trained (DT) and donor-sedentary (DS) mice as well as their recipient germ-free mice. We found 21 dominant genera in the microbiota profile from donor and recipient mice (relative abundance >2% in at least one group) (Figure 1A). The dominant genera were propagated from donors to recipients at week 1 and 8 in both sedentary and training conditions except for three unclassified genera belonging to the families *Porphyromonadaceae*, *Paludibacter*, and *Akkermansia* that were undetected in recipient mice. Chao1 index (amplicon sequence variant [ASV] richness estimation) and Shannon index (ASV evenness estimation) were used to compare the α-diversities and were found unaltered between DT and DS (Figures 1B and 1C). These indices were also similar between the recipient-trained (RT) and the recipient-sedentary (RS) mice (Figures 1B and 1C).

Our comparison between groups showed that a proportion of the three microbial genera showed more abundant trend, while a downward trend was observed in seven other genera (p < 0.1) (Table S1) in the DT group than in the DS. In the RT group, a proportion of seven genera showed a more abundant trend after 1 week than the RS group (Table S2). In contrast, at 1 week of fecal microbiota transplantation (FMT), there were 45 genera lower trend in abundance in RT mice (Table S2). At 8 weeks of FMT, a proportion of 11 genera were more abundant trend in RT mice than RS mice, while seven other genera were less abundant trend (Table S3). Within the dominant genera altered by training, two higher genera were commonly found in both donor and week 1-recipient mice: *Lactobacillus* (p < 0.05) and *Lactococcus* (p < 0.1) (Figure 1D, Tables S1 and S2). It is important to note that the two genera underwent more frequent alterations in their composition in DT and RT compared to DS and RS. Conversely, we indicate that the genus *Parabacteroides* is regularly decreased in DT (p < 0.1) and week-1 RT (p < 0.05) group (Figure 1E, Tables S1 and S2). Furthermore, at 1 week after FMT in RT group, we found a higher genus *[Ruminococcus]* belonging to the family *Lachnospiraceae* (p < 0.05) and two lower genera, an unclassified genus belonging to the family *Ruminococcaceae* in addition to an unclassified genus belonging to the order *Bacteroidales* (p < 0.05) (Figures 1D and 1E, Table S2).

### Ability of deconjugation of circulating BAs in recipient mice

**Figure 2:**
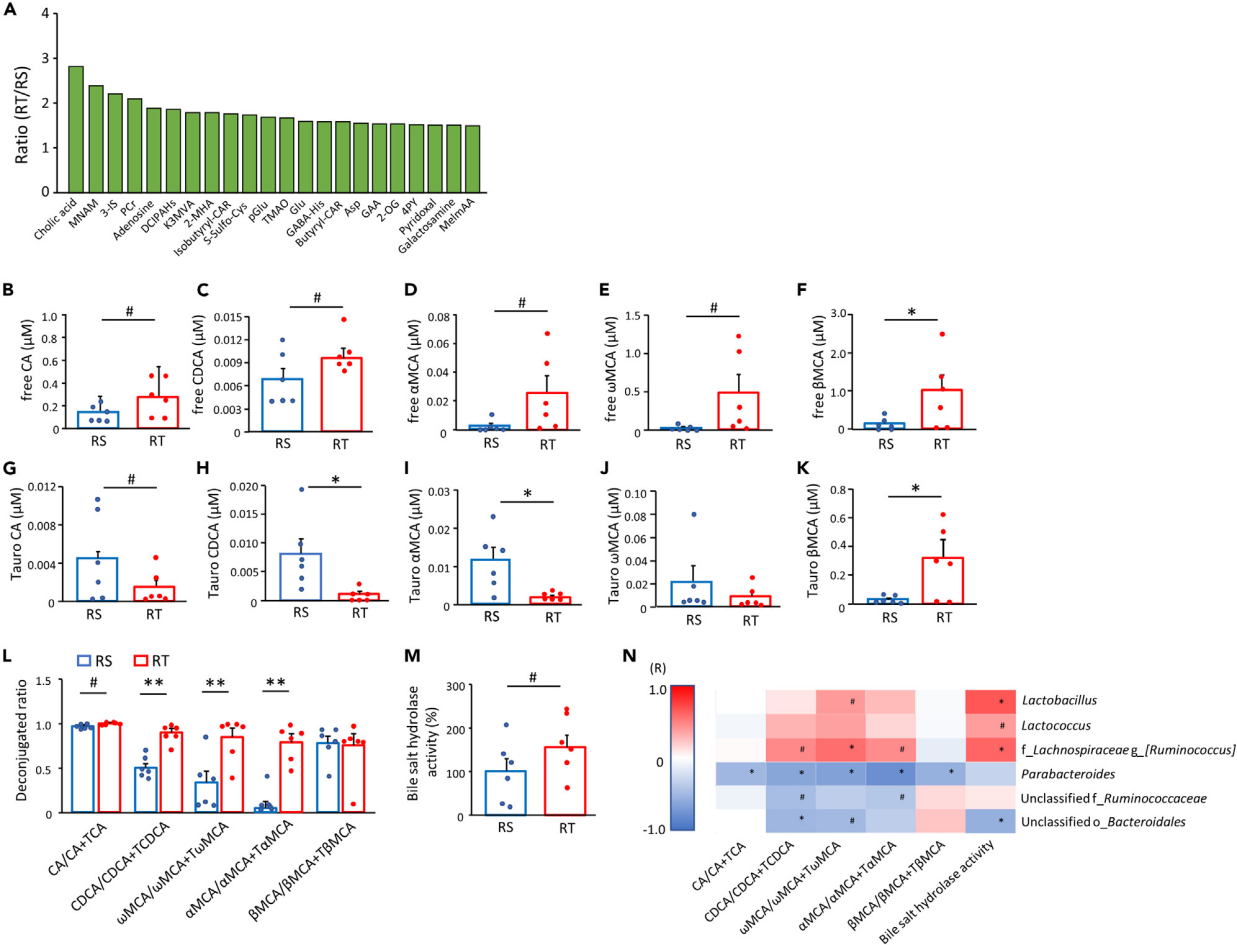
Deconjugation of plasma BA increased upon transplantation of exercise-acclimated fecal microbiota The plasma metabolites elevated by exercise-acclimated fecal microbiota (n = 3, A) and plasma free-form BAs (n = 6, B–F), tauro-conjugated BAs (n = 6, G–K), the deconjugated ratio (n = 6, L), and fecal bile salt hydrolase activity (n = 6, M) in recipient mice at week-1. Positive correlation of the deconjugation ratio of BAs with the presence of certain microbiota propagated from donors and bile salt hydrolase activity (n = 6, N) are shown in red, and negative correlations are shown in blue. MNAM; Methylnicotinamide, 3-IS; 3-Indoxylsulfuric acid, PCr; Phosphocreatine, DCIPAHs; 1H-Imidazole-4-propionic acid, K3MVA; Methyl-2-oxovaleric acid, 2MHA; 2-methylhippuric acid, Isobutyryl-CAR; Isobutyrylcarnitine, S-Sulfo-Cys; S-Sulfocysteine, pGlu; Pyroglutamic acid, TMAO; Trimethylamine N-oxide, Glu; Glutamic acid, GABA-His; γ-aminobutyryl-histidine, GAA; Guanidoacetic acid, 2-OG; 2-Oxoglutaric acid, 4PY; N^1^-Methyl-4-pyridone-5-carboxamide, MelmAA; 1-Methyl-4-imidazoleacetic acid, RS; recipient from sedentary donor, RT; recipient from trained donor. ^#^p < 0.1, ∗p < 0.05, and ∗∗p < 0.01. Results are presented as means ± standard errors.

Changes in the microbiota frequently affect various tissue functions in the host by impacting the circulating factors. Therefore, plasma metabolites obtained from recipient mice at 1 week after transplantation were analyzed using comprehensive metabolome analysis. In total, the levels of 22 factors were 1.5-fold higher in RT than in RS (Figure 2A, Table S4). Among them, cholic acid (CA), a representative primary bile acid (BA), showed the highest elevation. Hence, we focused our analysis on BAs and examined their profile.

Comprehensive BA analysis revealed that the free forms of CA, chenodeoxycholic acid (CDCA), α-muricholic acid (αMCA), and ωMCA, were higher in RT than in RS (Figures 2B–2F). In contrast, the levels of tauro-conjugated forms were lower in RT than in RS, except for ωMCA and βMCA (Figures 2G–2K). To examine BA conversion between the free and conjugated forms, we calculated the product/(product + substrate) to obtain the ratio of deconjugated BAs. We found a higher deconjugated ratio in RT than in RS for all examined BAs except for βMCA (CA, p = 0.059; CDCA, p < 0.01; ωMCA, p < 0.01; αMCA, p < 0.01) (Figure 2L), concomitant with a higher trend in bile salt hydrolase activity in the feces (p < 0.1) (Figure 2M). In addition, we found the deconjugated ratio to be positively correlated with the presence of the three more abundant genera in DT and RT at 1 week after FMT and negatively correlated with the three less abundant genera in DT and RT (Figure 2N). Bile salt hydrolase activity was also positively correlated with the presence of the three more abundant genera and showed a negative correlation with the abundance of an unclassified genus belonging to the order *Bacteroidales* (Figure 2N).

### Activation of metabolic signaling in skeletal muscle from recipient mice

**Figure 3:**
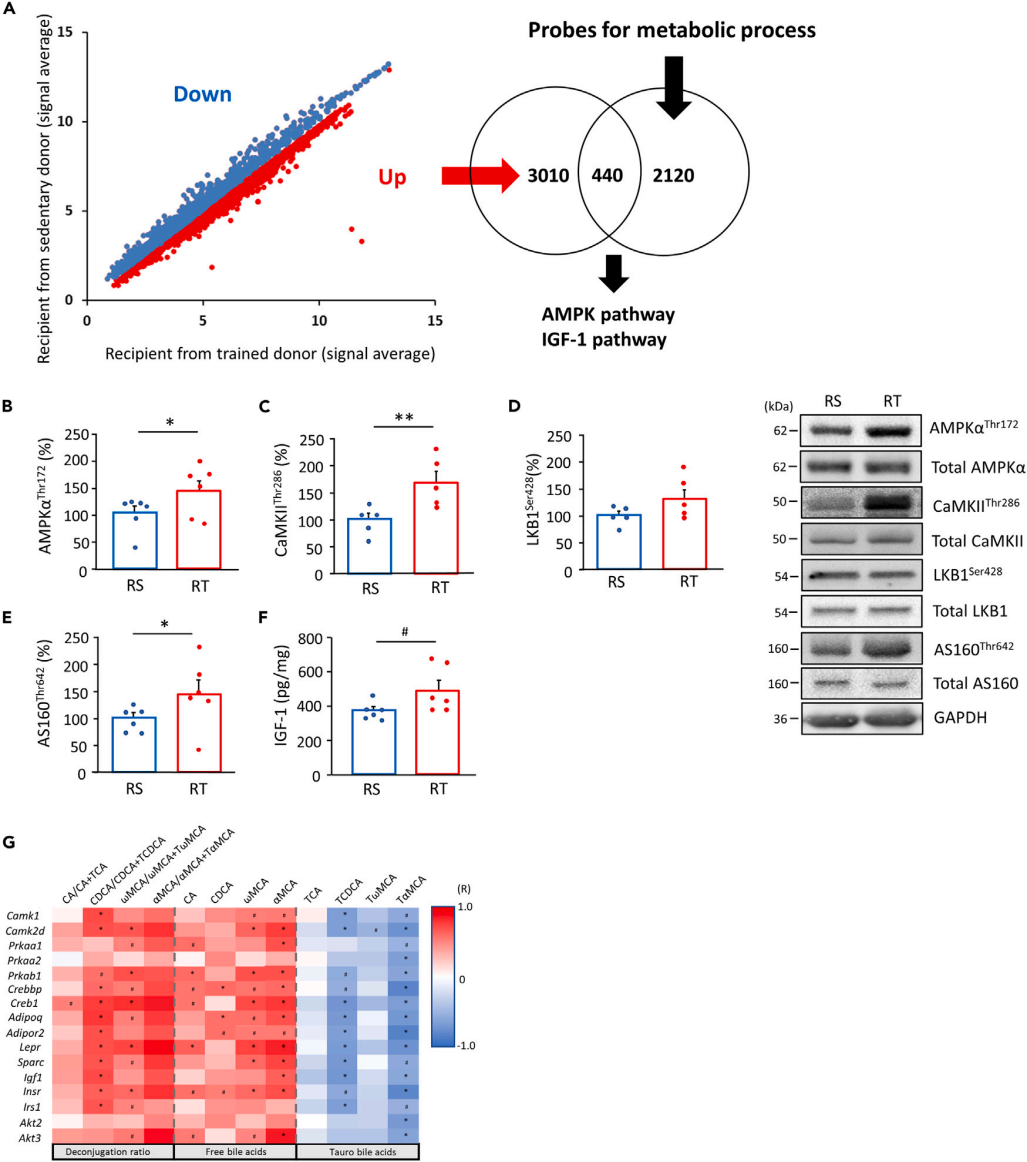
Upregulation of AMPK and IGF-1 in skeletal muscle by the transplantation of exercise-acclimated fecal microbiota A scatterplot of probes with significant differences (p < 0.05, FDR <0.1) between both the recipient mice at week-1 (n = 6, A). mRNA levels of 3,450 genes were significantly higher in recipient mice transplanted with exercise-acclimated fecal microbiota than those in mice transplanted from sedentary donors. Of these, 440 genes were categorized in energy metabolic processes, including “glucose metabolic process,” “carbohydrate metabolic process,” “lipid metabolic process,” “glycerol metabolic process,” “ATP catabolism,” “tricarboxylic acid cycle,” and “oxidation-reduction process.” Ingenuity canonical pathway analysis indicated that AMPK and IGF-1 signals were positively regulated by FMT from trained donors. AMPKα^Thr172^ (B), CaMKII^Thr286^ (C), LKB1^Ser428^ (D), and AS160^Thr642^ (E) phosphorylation and IGF-1 content (F) in gastrocnemius muscle from recipient mice (n = 5–6). The correlation of plasma BA profile with muscle metabolic factor level in recipients is shown (n = 6, G). High levels of important factors related to AMPK and IGF-1 signaling in the skeletal muscle of recipient mice in transcriptome analysis correlates with free-, tauro-BAs, and deconjugation ratios of BAs. Positive correlations are shown in red, and negative correlations are shown in blue. The correlation coefficient was calculated using Spearman’s correlation analysis. Phosphorylation levels were correlated with total content of each target in immunoblotting. RS; recipient from sedentary donor, RT; recipient from trained donor. ^#^p < 0.1, ∗p < 0.05, and ∗∗p < 0.01. Results are presented as means ± SE.

A typical adaptive change of habitual exercise is the metabolic improvement of the skeletal muscle.^5^ Hence, we examined the metabolic factors in the skeletal muscle of recipient mice using transcriptome analysis. Microarray analysis revealed that the expression of 3,450 genes was higher and that of 3,765 genes was lower in the gastrocnemius muscle of week 1-RT (Figure 3A). Of the upregulated genes, 440 were related to energy metabolic process, including “glucose metabolism”, “carbohydrate metabolism”, “lipid metabolism”, “glycerol metabolism”, “ATP catabolism”, “tricarboxylic acid cycle”, and “oxide-reduction process” as per the Gene Ontology Biological Process Term analysis. Ingenuity canonical pathway analysis revealed that AMPK and insulin growth factor-1 (IGF-1) signaling were highly ranked in pathways activated by FMT from DT (Figures 3A, S1, and S2). We also found positive correlations between factors related to AMPK and IGF-1 signaling and the deconjugated ratios, with higher levels present in RT mice (Figure 3G). Based on these findings, we next determined whether FMT altered the key factors related to those signaling pathways in skeletal muscle. AMPKα^Thr172^ phosphorylation was increased in the skeletal muscles of RT (p < 0.05) (Figure 3B). In addition, the phosphorylation of CaMKII^Thr286^, an upstream regulator of AMPK, was increased (p < 0.01) in RT mice (Figure 3C). Conversely, phosphorylation of liver kinase B1 (LKB1)^Ser428^, another upstream regulator, was unaltered by FMT from DT (Figure 3D). Phosphorylation of AS160^Thr642^, a downstream factor of AMPK, was also higher in RT (p < 0.05) (Figure 3E). A network analysis of the transcriptome, plasma BAs, and the abundance of bacterial genera revealed that *Lactobacillus*, *Lactococcus*, and [*Ruminococcus*] affected AMPK signaling factors in the BA-dependent or independent routes (Figure S3). IGF-1 levels also trended higher in the RT mice (p = 0.058) (Figure 3F).

AMPK is a major exercise-inducible metabolic regulator in skeletal muscle. To examine the relationship between muscle AMPK activity and the intestinal microbiota, the microbiota profile of AMPKγ3-knockout mice was obtained, since AMPKγ3 is predominantly expressed in skeletal muscle.^19^ At the genus level, AMPKγ3-knockout mice showed a trend toward lower abundance for four genera, namely *[Ruminococcus]*and unclassified genera belonging to the family *Lachnospiraceae, Clostridium*, and *Butyricicoccus* compared to wild-type mice (p < 0.1) (Figure S4). Although the profile did not match recipient mice, the changes in *[Ruminococcus]* and the unclassified genus belonging to the family *Lachnospiraceae* were opposite to those in RT mice. In contrast, higher abundance of genera in AMPKγ3-knockout mice was not found.

### Different cholic acid forms alter inflammatory and metabolic responses in cultured myotubes and muscle tissues

**Figure 4:**
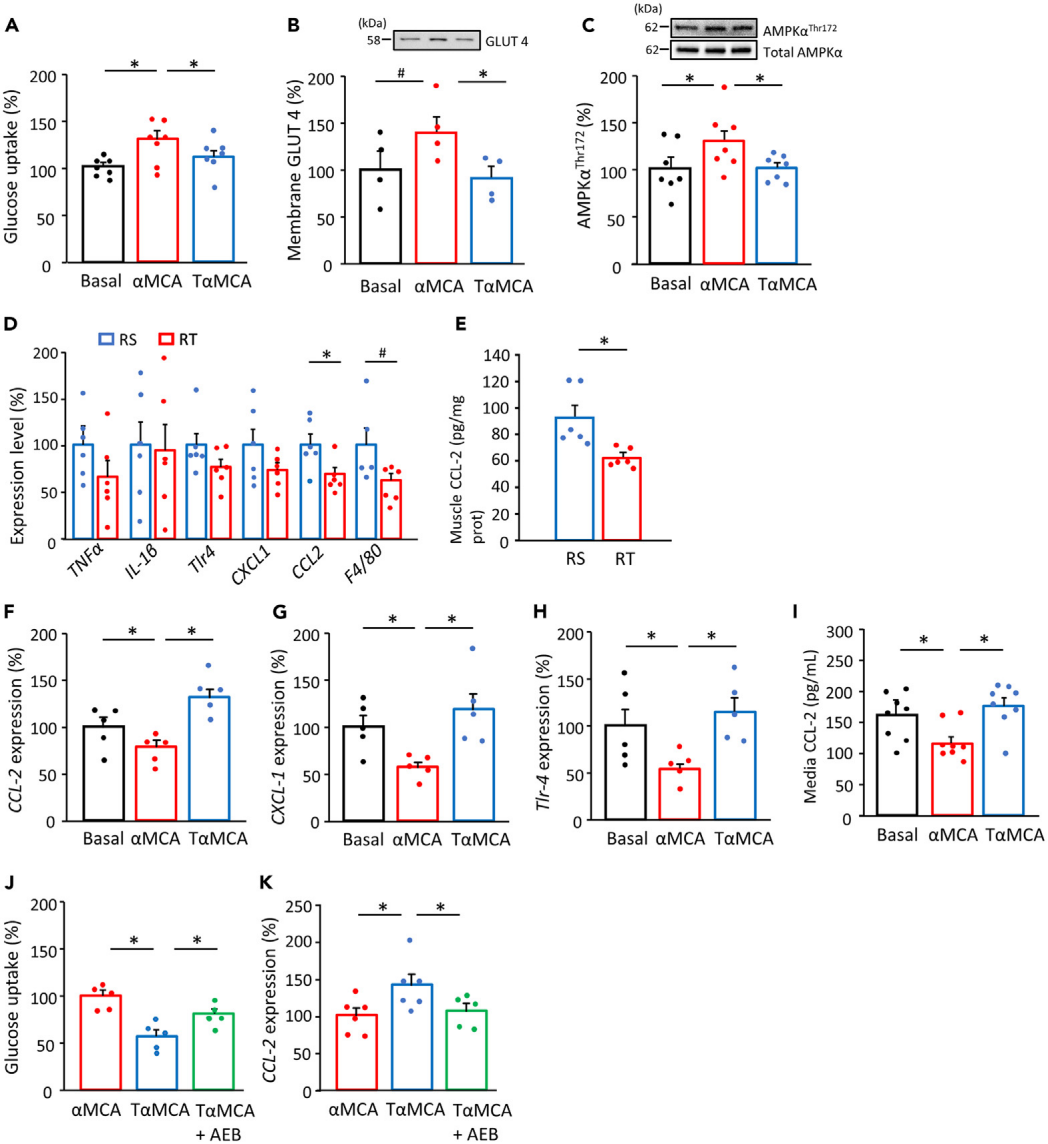
Different forms of BA modulate metabolic and inflammatory responses in cultured myotubes and muscle tissues Glucose uptake in C2C12 myotubes incubated in the absence or presence of αMCA and tauro α-muricholic acid (TαMCA) (10 μM) with palmitic acid (200 μM) for 24 h or incubated with or without sotrastaurin (AEB071) (2 nM) (n = 5–8, A and J). The membrane content of glucose transporter 4 (GLUT4) (n = 4, B) and AMPKα^Thr172^ phosphorylation (n = 7, C) in C2C12 myotubes incubated in the absence or presence of MCA and TMCA. mRNA levels of *CCL-2*, *CXCL-1*, and *Tlr-4* (n = 5–6, F–H, K) with or without AEB071in myotubes, and levels of CCL-2 in the media (n = 8, I) in the absence or presence of αMCA and TαMCA. mRNA levels of *TNF-α*, *IL1-β*, *Tlr-4*, *CXCL-1*, and *CCL-2*, and *F4/80* (n = 6, D) and CCL-2 protein level (n = 6, E) in gastrocnemius muscles from recipient mice at week-1. Phosphorylation levels were correlated with the total content of each target in immunoblotting. RS; recipient from sedentary donor, RT; recipient from trained donor. ^#^p < 0.1 and ∗p < 0.05 between groups. Results are presented as the mean ± SE.

The effect of free and tauro-conjugated αMCAs, the highest deconjugated BA, was examined in a palmitate-induced insulin-resistant culture experiment model that showed lower insulin signaling and higher expression of inflammatory factors in C2C12 myotubes (Figures S5A–S5D). Glucose uptake was elevated by treatment with free-form αMCA in the presence of palmitic acids (p < 0.05) (Figure 4A). Along with higher content of glucose transporter 4 (GLUT4) in the membrane fraction (Figure 4B), free-form αMCA treatment also increased the AMPK^Thr172^ levels (p < 0.05) (Figure 4C). By contrast, elevated glucose uptake and AMPK activation were not observed following treatment with tauro-conjugated αMCA, which is supporting evidence for a direct effect of BAs. In our experiment with different content ratios of the two BA forms, the medium with higher free-form also showed higher activation of AMPK signaling than the medium with increased tauro-conjugated form (Figure S6A and S6B). However, the IGF-1 levels were comparable between the media with free and conjugated BA forms (Figure S7).

Inflammatory factors are involved in metabolic impairment. Typical inflammatory factors in muscle tissues were measured to examine the mechanism of BA deconjugation on metabolic signaling activation. We found lower expression levels of chemokine (C-C motif) ligand 2 (CCL-2) (p < 0.05) and F4/80 (p = 0.067) in the muscle tissues of RT at 1 week after FMT (Figures 4D and 4E), suggesting suppressed macrophage infiltration, a marker of low-grade inflammation. In cultured myotubes, the mRNA expression of inflammatory factors was higher upon treatment with tauro-conjugated BA than with the free-form (p < 0.05) (Figures 4F–4H) whereby CCL-2 concentration in media was also higher (p < 0.05) (Figure 4I). Sotrastaurin, an inhibitor of protein kinase C-theta (PKCθ), which is a possible factor of the inflammatory pathway in response to BA, prevented the effects of tauro-conjugated form on glucose uptake and CCL-2 levels (p < 0.05) (Figures 4J and 4K).

### Transplanting exercise-induced microbiota improves glucose metabolism in high-fat diet-induced obese mice

**Figure 5:**
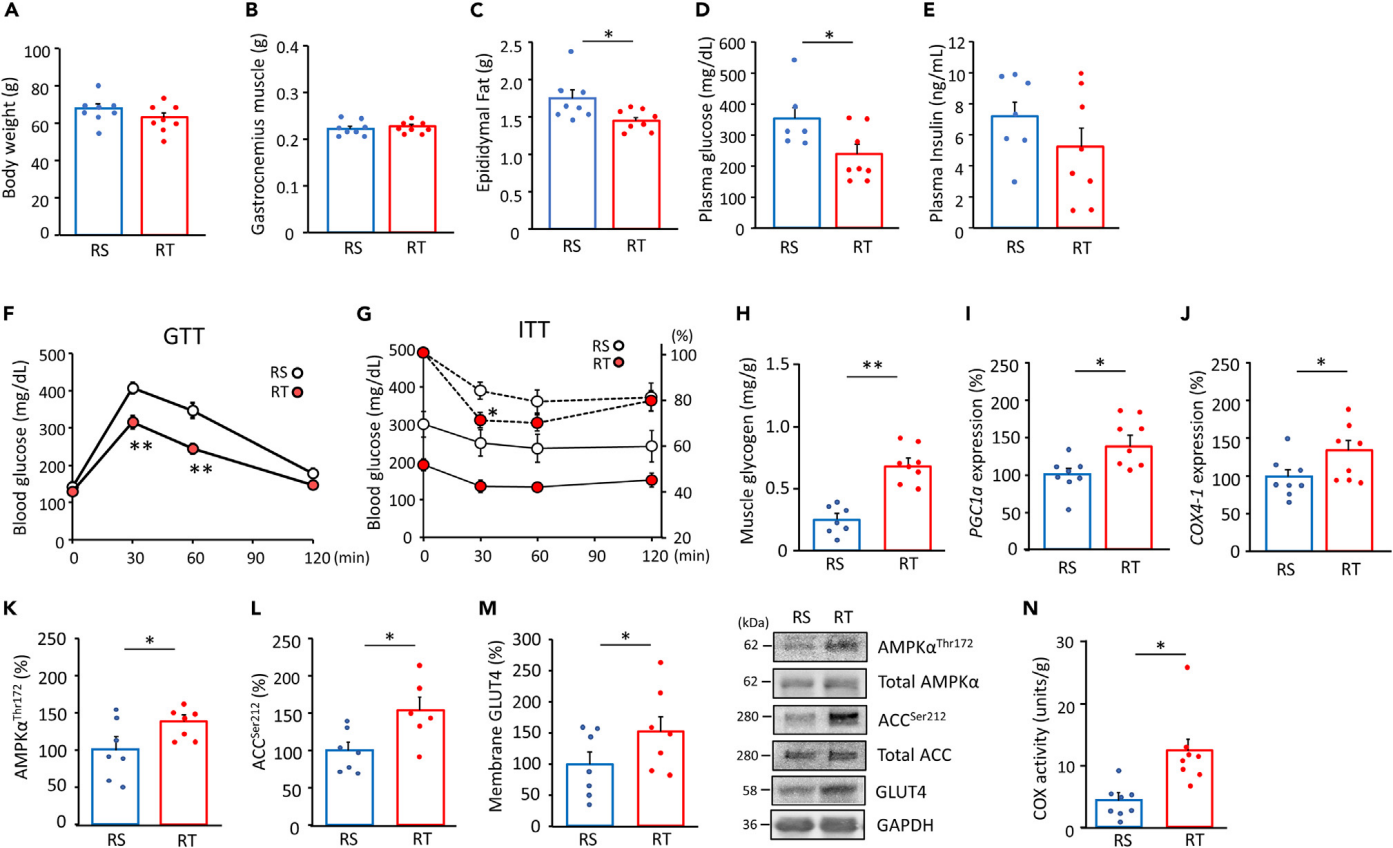
Exercise-acclimated microbiota treatment improves glucose tolerance in HFD-fed mice (AN) Body and tissue weights (n = 8, A–C), blood chemistry (n = 8, D and E), blood glucose concentrations during oral glucose tolerance tests (n = 8, GTT) (F) and insulin tolerance tests (ITT) (n = 8, G), glycogen content (n = 7–8, H), mRNA levels of *PGC1α* and *COX4-1* (n = 8, I and J), AMPKα^Thr172^ (n = 7, K) and ACC^Ser212^ (n = 6–7, L) phosphorylation, membrane content of GLUT4 (n = 7, M), and COX activity (n = 8, N) in gastrocnemius muscle in recipient mice fed chow or HFD for 8 weeks. Phosphorylation levels were correlated with total content of each target in immunoblotting (K–M). The solid line shows absolute values, and the dotted line shows relative values in the ITT (G). RS; recipient from sedentary donor, RT; recipient from trained donor. ∗p < 0.05, and ∗∗p < 0.01 between groups. Results are presented as the mean ± SE.

The effects of FMT on glucose metabolism were determined in high-fat diet (HFD)-induced obese mice. After taking HFD, body weight, blood glucose, and plasma insulin were gradually increased over 8 weeks (Figures S8A–S8C), in addition to decreased level of phopho-Akt in gastrocnemius muscle (Figure S8D). Body weight did not change between recipient groups after taking HFD for 8 weeks (Figure 5A). Although the gastrocnemius muscle weight was unaltered, RT mice showed lower epididymal fat weight (p < 0.05) (Figures 5B and 5C), concomitant with lower plasma glucose (p < 0.05) and unaltered insulin levels (Figures 5D and 5E). Blood glucose levels during oral glucose tolerance test (GTT) were lower in RT mice (30 min and 60 min, p < 0.01) (Figure 5F). Insulin tolerance test (ITT) showed gradual decrease in blood glucose levels after insulin injection, and the relative decrease rate was larger in RT mice (30 min, p < 0.05) (Figure 5G). These improvements in metabolic parameters in HFD-induced obese mice correlated with enhanced glycogen content, mRNA expressions of peroxisome proliferator-activated receptor gamma coactivator-1α (PGC1α), cytochrome *c* oxidase 4-1 (COX4-1), and AMPKα^Thr172^, acetyl-CoA carboxylase (ACC)^Ser212^ phosphorylation, and membrane content of GLUT4, as well as COX activity in the gastrocnemius muscle of RT mice compared to the RS group (p < 0.05) (Figures 5H–5N).

## Discussion

In this study, we focused on the effect of gut microbiota transferred from trained to recipient mice via FMT, on the development of metabolic dysfunction induced by HFD. Given the characteristics of the mouse model, bacterial and molecular changes in the early phase of HFD feeding may be involved in glucose intolerance and obesity in later phases. Hence, we conducted transcriptome and metabolome analysis at 1 week of FMT and examined phenotypic parameters at week 8. We found that recipient mice transplanted with gut bacteria from DT mice showed reduced HFD-induced glucose intolerance. In the recipient mice, the glucose metabolic signaling pathway in skeletal muscle was activated with a higher ability of deconjugation of BA, which is involved in metabolic and anti-inflammatory responses. These results suggest that exercise-induced gut microbial modulation contributes to improved glucose metabolism in skeletal muscle through BA deconjugation. Furthermore, our findings confirm that bacterial composition in the gut influences the organ functions throughout the body.

Cross-sectional and interventional studies in animals and humans show that habitual exercise alters the composition of the intestinal microbiota.^8^^,^^9^ However, it is unclear whether microbiota alterations contribute to exercise-induced metabolic improvements. In the present study, we observed an increase in the abundance of the common bacterial genera *Lactobacillus* and *Lactococcus* in both DT and RT mice. These microbes are frequently more abundant in exercise habituation^20^^,^^21^ and less abundant in metabolic disorders in animals and humans^22^^,^^23^ whereby dietary supplementation with *Lactobacillus* and *Lactococcus* improves glucose tolerance.^24^^,^^25^ Conversely, we report a regular decrease of the genus *Parabacteroides* that is commonly elevated in obesity as well as in metabolic dysfunction^22^^,^^23^ and is decreased by exercise.^26^ In addition to these commonly altered genera, 44 microbial species showed lower presence, while 5 were higher in RT mice. FMT to germ-free mice is a typical model used to promote the propagation of microbes and evaluate their function; however, recipients do not yield an entire similar microbiota profile as the donor. Furthermore, HFDs may also interfere with the propagation of microbiota in the recipients as it is established that a daily HFD causes dysbiosis, associated with metabolic dysfunction.^27^ By contrast, the metabolic benefits of exercise were better obtained in the HFD group than in the normal-diet group.^28^ Therefore, exercise-induced changes in these genera may contribute to metabolic improvement under HFD conditions.

One of the crucial benefit of habitual exercise is improved metabolic capacity, such as insulin sensitivity and mitochondrial oxidation.^5^ Because skeletal muscle is the principal site for blood glucose disposal, it is a practical target in therapies to prevent and treat obesity and type 2 diabetes. In the present study, we found that various metabolic activators, including enzymes, transcription factors, and myokines, were upregulated in the skeletal muscle in RT mice. Particularly, AMPK signaling was activated following FMT from DT. AMPK is a major sensor of intracellular energy demand that stimulates glucose uptake and mitochondrial biogenesis.^29^ It activates insulin-independent glucose uptake and lipid oxidation in skeletal muscle in response to exercise and muscle contraction.^29^^,^^30^^,^^31^ In contrast, it has been suggested that a permanent reduction in the AMPK pathway leads to insulin resistance^32^ and contributes to muscle dysfunction in diet-induced obesity.^33^ Our results showed that phospho-AMPK and phospho-AS160 levels were elevated in the skeletal muscle of the RT mice. In addition, because one of the primary insulin target organs is skeletal muscle, our GTT and ITT results support a beneficial improvement in skeletal muscle glucose uptake. Hence, these findings indicate that the alterations in metabolic functions as a result of FMT involve AMPK signaling in skeletal muscle. Although the genera affected by AMPKγ3 knockout did not necessarily match the genera that were influenced by FMT, the lower genus *[Ruminococcus]* in AMPKγ3-knockout mice corresponds to the higher result in RT mice. However, because the whole composition rather than changes in individual bacteria affects the host, further studies are needed in order to corroborate present results and confirm whether the exercise-induced change in microbiota can be induced by AMPK activation in skeletal muscle.

While AMPK phosphorylation is reduced in skeletal muscle derived from animal models of obesity or type 2 diabetes,^34^ humans with type 2 diabetes retain AMPK activation in response to exercise or cellular stress.^35^ Thus, agents that mimic the effects of exercise on AMPK activation may improve insulin sensitivity of skeletal muscle in metabolic diseases. Our results show that gut microbiota from trained mice prevents metabolic disruption in recipient mice under HFD conditions. Therefore, long-term FMT treatment may enhance skeletal muscle insulin sensitivity via AMPK activation, thereby preventing the development of type 2 diabetes. Furthermore, our results suggest that gut microbiota are involved in improved skeletal muscle metabolism during exercise training, which partly explains the health-promoting effects of physical activity in the prevention of peripheral insulin resistance. Because the elevated glycogen content leads to the enhancement of endurance capacity,^36^^,^^37^ the exercise-acclimated microbiota may also have a potential benefit for athletic performance. However, the main objective of this study was to examine the effect of FMT on diet-induced metabolic dysfunction; thus, appropriate experiment protocol was not set up to assess endurance.

Several microbiota-produced metabolites play critical roles in immune and metabolic functions,^12^^,^^38^ namely, short-chain fatty acids, trimethylamine, amino acids, hydrogen peroxides, and lactate,^12^^,^^38^^,^^39^^,^^40^ which are released into the circulation. In metabolome analysis of circulating metabolites, we found that CA was the maximum enhanced factor in the RT group compared to the RS group. Primary BAs such as CA and CDCA are generated and conjugated with glycine or taurine (Glyco-CA, Glyco-CDCA, Tauro-CA, Tauro-CDCA) in the liver. These primary BAs are exported into the bile, where glycine and taurine are deconjugated by specific bacteria that express bile salt hydrolases, such as *Lactobacillus* and *Bifidobacterium*.^41^ In mice, MCAs are the primary BAs that are conjugated with glycine and taurine and deconjugated by bacteria. Accumulating evidence suggests that BAs can regulate nutrient metabolism by regulating the activation of the BA-specific receptors, farnesoid X receptor and transmembrane G protein-coupled receptor (TGR)-5, in metabolic tissues.^42^ We found that plasma taurine-conjugated BA levels were decreased and free-form BA levels were increased in HFD-fed RT mice, which was confirmed by a higher deconjugation ratio. Incidentally, the deconjugation ratio showed a positive correlation with increased bacterial genera in both DT and RT mice, supporting the relationship between microbiota and circulating modifications in BAs, in particular, the genus *Lactobacillus* as it has a high activity of bile salt hydrolase.^41^^,^^43^ A previous study also showed that HFDs increase the total level of taurine-conjugated BAs and decrease the level of free BAs.^44^ Taurine-conjugated BAs have been suggested to induce higher inflammatory responses than free BAs in adipocytes, which is associated with increased CCL-2 expression.^45^ Therefore, exercise-induced microbiota changes suppressed metabolic dysfunction caused by HFDs, which may be partly mediated by deconjugated BAs. In cultured myotubes, we found that free MCA treatment activated glucose uptake and AMPK signaling more than the treatment with tauro-conjugated MCA. Network analysis also supported the relationship between the microbiota, BA deconjugation, and AMPK signaling. βMCA did not show a higher deconjugation ratio in the RT mice or a correlation of the deconjugation ratio with metabolic activators in skeletal muscle. In contrast to other MCAs, it has been reported that the tauro-conjugation form of βMCA protects against tissue damage and induces metabolic improvement.^46^ Hence, the higher tauro-conjugated form of βMCA may contribute to metabolic activation in the muscle.

Recently, several studies have examined exercise effects using the FMT approach. Zoll et al.^47^ showed that the metabolic-disrupting effect of high-fat and high-sugar diet was propagated from donors to recipients fed with normal diet weekly by FMT. However, this did not elucidate the benefits of exercise training. In contrast, Lai et al.^48^ reported the transmission of training-induced metabolic improvement in donors by higher frequency (5 times/week) FMT on recipients fed with HFD after bacterial elimination with antibiotic treatment. This result indicated that exercise-acclimated microbiota can contribute to improved metabolic dysfunction under HFD conditions. Meanwhile, the present study showed that even lower frequency (twice weekly) of FMT to germ-free mice fed with HFD successfully achieved the advantageous metabolic effects. Germ-free conditions in the initial state, diet, and FMT method may have led to a more efficient transmission. Furthermore, the training program and the type of donor mice may also affect the gut microbiota profile and efficient transmission to the recipients. In particular, Institution of Cancer Research (ICR) mice can perform high-intensity exercise regime better compared to B6J and Balb/c mice; hence, sufficient training load may have also influenced the results.

Various inflammatory factors impair glucose metabolism in skeletal muscle. Animal models and patients with type 2 diabetes show higher inflammatory cytokine levels,^49^ and chronic low-grade inflammation causes insulin resistance.^50^ Our results showed a lower expression of CCL-2 and F4/80, a macrophage infiltration marker, in the RT mice in the early period of FMT, i.e., the pre-stage onset of glucose intolerance. Furthermore, tauro-conjugated BA treatment also resulted in more CCL-2 expression and secretion than treatment with free-form BA in cultured myotubes. CCL-2 suppresses macrophage-dependent and independent glucose uptake.^49^^,^^51^ Therefore, these observations suggest that CCL-2 mediates taurocholic acid-induced suppression of glucose uptake via an inflammatory response. Although we observed the association of deconjugation with the abundance of bacterial genera, a detailed analysis with bacterial species class would have provided more insights.

Generally, circulating BAs act as signaling factors through farnesoid X receptor and TGR-5. Particularly in skeletal muscle, TGR-5 mediates signal activation in protein anabolism and energy utilization.^52^^,^^53^ BA composition does not lead to a large difference in TGR-5-mediated metabolic signaling.^52^^,^^53^ However, the present results are not necessarily limited to TGR-5-mediated effects but rather suggest that the induction of inflammatory factors such as CCL2 attenuates glucose metabolic signals. Given that CCL2 is induced by conjugated BA in adipocytes,^45^ ability to deconjugate may contribute to metabolic improvement via attenuated inflammatory responses. BAs can directly disrupt the plasma membrane and activate the protein kinase C (PKC) pathway, which results in inflammatory responses.^54^^,^^55^ In skeletal muscle, inflammatory responses are mediated by activated PKCθ.^56^^,^^57^ We found that pretreatment with PKCθ inhibitor prevented taurocholic acid-induced suppression of glucose metabolism and elevated CCL-2. Collectively, our findings suggest that exercise-acclimated microbiota improves glucose tolerance by suppressing the tauro-conjugated BA-induced inflammatory response. BAs can regulate intracellular calcium levels.^58^ Because AMPK phosphorylation is activated by calcium-dependent signals,^59^ intracellular calcium may also be involved in metabolic improvement. By contrast, IGF-1 signaling, another predicted signaling pathway activated by FMT, did not change between free and conjugated BAs. As shown in a previous study,^52^ this signaling is activated through TGR-5, which occurs via both free and conjugated BAs. Although the different responses of IGF-1 observed between RS and RT are unclear, we speculate that other microbiota-responsive factors may have mediated this effect. However, further studies employing various conditions in ratios, concentrations, and species of BAs would be required to provide more insight. Apart from BAs, other metabolite levels can be increased by transplanting microbiota from DT, which in turn may have several metabolic benefits and other multiple effects. In addition to short-chain fatty acids and organic acids suggested in previous studies,^38^^,^^40^ metabolites found in the present metabolome analysis may be potential factors. As this study only demonstrated the effect of BAs as a contributing factor, more research on the mechanism of metabolic action in skeletal muscle is necessary to fully determine the effect of exercise-induced microbiota changes. Furthermore, it is necessary to confirm the potential application of these findings in human studies. In addition to the concept of fecal transplantation from trained subjects, the consumption of probiotics with high bile salt hydrolases activity may provide metabolic benefits.

In conclusion, microbiota transplantation of feces from trained mice plays a role in whole-body glucose tolerance, concomitant with AMPK activation in skeletal muscle. These effects are mediated by anti-inflammatory properties of BA deconjugation. Microbiota transplantation improves glucose homeostasis and insulin sensitivity in HFD-induced obese mice, which highlights the metabolic benefits obtained by habitual exercise and FMT application in the management of metabolic disease. Thus, we concluded that microbiota mimics the health-promoting effects of exercise by improving glucose metabolism in skeletal muscle through the “muscle and gut axis.”

### Limitations of the study

Although our study provides a crucial link between the influence of exercise on microbiota and in turn its effect on the metabolic functions of skeletal muscles, there are a few limitations. As mentioned previously, the effects of the whole composition of the microbiota on metabolism could be much broader than those observed upon altering a small proportion. Therefore, further studies are requried to address this on a larger scale. Moreover, although we have found the correlation between AMPK signaling and the abundance of certain bacterial genera, further studies are required to establish a mechanistic link between the two. More importantly, based on the metabolome analysis, we focused our study on the BAs. Further experiments are required to elucidate the mechanistic details of microbiota-mediated metabolic regulation in the skeletal muscle. Furthermore, the effect of other microbiota-released metabolites must also be studied in this context.

## STAR★Methods

### Key resources table

**Table:** 

REAGENT or RESOURCE	SOURCE	IDENTIFIER
**Antibodies**		

Rabbit polyclonal anti-phospho AMPKα (Thr172)	Cell Signaling Technology	Cat#2531S; RRDI: AB_330330
Rabbit polyclonal anti-AMPKα	Cell Signaling Technology	Cat#2532S; RRID: AB_330331
Rabbit polyclonal anti-phospho ACC (Ser212)	Cell Signaling Technology	Cat#3661S; RRID: AB_330337
Rabbit polyclonal anti-ACC	Cell Signaling Technology	Cat#3662S; RRID: AB_2219400
Rabbit polyclonal anti-phospho Akt (Ser473)	Cell Signaling Technology	Cat#9271S; RRID: AB_329825
Rabbit polyclonal anti-Akt	Cell Signaling Technology	Cat#9272S; RRID: AB_329827
Rabbit polyclonal anti-phospho Akt substrate (Ser/Thr)	Cell Signaling Technology	Cat#9611S; RRID: AB_330302
Rabbit polyclonal anti-phospho AS160 (Thr642)	Cell Signaling Technology	Cat#8881S; RRID: AB_2651042
Rabbit polyclonal anti-AS160	Cell Signaling Technology	Cat#2670S; RRID: AB_2199375
Rabbit polyclonal anti-phospho CaMKII (Thr286)	Cell Signaling Technology	Cat#12716S; RRID: AB_2713889
Rabbit polyclonal anti-CaMKII	Cell Signaling Technology	Cat#4436S; RRID: AB_10545451
Rabbit polyclonal anti-phospho LKB1 (Ser428)	Cell Signaling Technology	Cat#3482S; RRID: AB_2198321
Rabbit polyclonal anti-LKB1	Cell Signaling Technology	Cat#3047S; RRID: AB_2198327
Rabbit polyclonal Anti-GLUT-4 C-terminus	Merck Millipore	Cat#07-1404; RRID: AB_1587080
Mouse monoclonal Anti-GAPDH	Abcam	Cat#ab8245; RRID: AB_2107448

**Biological samples**		

Mouse skeletal muscle	This paper	N/A
Mouse blood	This paper	N/A
Mouse stool	This paper	N/A

**Chemicals, peptides, and recombinant proteins**		

αMCA	Cayman	Cat#20291
Tauro-αMCA	Cayman	Cat#20288
Tauro-αMCA	Toronto Research	Cat#T009130
Sotrastaurin	Selleck	Cat#S2791

**Critical commercial assays**		

Mouse MCP-1/CCL2 ELISA kit	Sigma	Cat#RAB0055-1KT
Mouse IGF-1 ELISA kit	Protein tech	Cat#KE10032
Mouse Insulin ELISA kit	Mercodia	Cat#10-1247-01
Glucose Uptake-Glo Assay	Promega	Cat#J1341
Proteoxtra Transmembrane Protein Extraction Kit	Novagen	Cat#71772-3CN
F-kit glucose	Roche Diagnostics	Cat# 716251A
Taqman Gene Expression Assay CCL-2	Thermo Fisher Scientific	ID#Mm00441242_m1
Taqman Gene Expression Assay CXCL-1	Thermo Fisher Scientific	ID#Mm04207460_m1
Taqman Gene Expression Assay TLR-4	Thermo Fisher Scientific	ID#Mm00445273_m1
Taqman Gene Expression Assay TNF-α	Thermo Fisher Scientific	ID#Mm00443258_m1
Taqman Gene Expression Assay IL-1β	Thermo Fisher Scientific	ID#Mm00434228_m1
Taqman Gene Expression Assay F4/80	Thermo Fisher Scientific	ID#Mm00802529_m1
Taqman Gene Expression Assay COX4-1	Thermo Fisher Scientific	ID#Mm01250094_m1
Taqman Gene Expression Assay PGC1-α	Thermo Fisher Scientific	ID#Mm01208835_m1
Taqman Gene Expression Assay β-actin	Thermo Fisher Scientific	ID#Mm 00607939_s1

**Deposited data**		

Raw and analyzed data	This paper	GEO: GSE201202

**Experimental models: Cell lines**		

Mouse: C2C12 cells	ECA	Cat#EC91031101-F0

**Software and algorithms**		

QIIME2	Bolyen et al., 2019^60^	Version 2020.8
Cytoscape	Shannon et al., 2003^61^	Version3.8.2
Transcriptome Analysis Console Software	Affymetrix	Version 4.0
Ingenuity Pathway Analysis	QIAGEN	Version 76765844
ImageJ	Schneider et al., 2012^62^	https://imagej.nih.gov/ij/

**Other**		

MiSeq	Illumina	Cat#SY-410-1003
Agilent time-of-flight mass spectrometer	Agilent Technologies	ID#6210
SpectraMax microplate reader	Molecular Devices	SpectraMac i3x
StepOne Plus Real-Time PCR system	Life Technologies	Cat#4376598
Bond Elut C18 cartridge	Agilent Technologies	Cat#12102025

### Resource availability

#### Lead contact

Further information and requests for resources and reagents should be directed to and will be fulfilled by the lead contact, Wataru Aoi (waoi@kpu.ac.jp).

#### Materials availability

This study did not generate unique new reagents or mouse lines.

### Experimental model and subject details

#### Animal studies

Animal studies were performed according to the guidelines of the Japanese Council on Animal Care and the Regulations and General Advice of Laboratory Animals of the Swedish Board of Agriculture. All animal experiments were approved by the Committees for Animal Research of Kyoto Prefectural University, Kyoto Prefectural University of Medicine (KPU20200402-R, M25-39) (Kyoto, Japan), and by the Stockholm Ethical Committee (Stockholm, Sweden).

Male Institution of Cancer Research (ICR) mice were used as donors to obtain feces. Mice were acclimatized in an air-conditioned (22 ± 2°C) room with a 12:12 h light–dark cycle. The mice in the exercise group ran on a motorized treadmill five times per week for four weeks. During this period, the level of exercise was gradually increased from running for 20 min at 18 m·min^−1^ to 60 min at 30 m·min^−1^. Fresh fecal samples were collected the day after the last exercise session. The mice were then euthanized, and samples of the gastrocnemius muscle and plasma were collected. The feces were immediately weighed and diluted 10-fold with sterile phosphate buffer that was pre-deoxygenated under an argon gas stream. The suspension was centrifuged (100 × *g*, 2 min, 4 °C), and the upper layer was collected and mixed with sterile 10% glycerol for FMT. The samples were dispensed into vials, immediately frozen in liquid nitrogen, and stored at −80°C until further use.

On arrival, twenty-eight male germ-free mice (10 weeks old, Sankyo Labo Service, Tokyo, Japan) were divided into two groups and administered fecal samples from sedentary or training donor mice by gavage under germ-free conditions. Then, the recipient mice were kept under specific-pathogen-free conditions and allowed free access to autoclaved water and a laboratory HFD (Research Diets, New Brunswick, NJ) containing protein, 20% kcal; fat, 45% kcal; and carbohydrate, 35% kcal. FMT was conducted twice a week throughout the housing period. The mice were housed together in cages with filtered tops and received identical fecal samples. After 1 week, six mice from each recipient group were fasted for 4 h. The mice were then euthanized, and muscle tissues, blood, and fecal samples were collected, immediately frozen, and stored at −80°C. For the remaining eight mice of each recipient group, oral GTT and intraperitoneal ITT were performed at week 8. Subsequently, muscle tissues and plasma were collected for biochemical assays.

Male AMPK γ3-knockout mice were generated using gene-targeting techniques.^63^ Mice were acclimatized in an air-conditioned (22 ± 2 °C) room with a 12:12 h light–dark cycle. Both knockout and wild-type littermate mice were used for high throughput 16S rRNA amplicon sequencing of fecal samples collected after fasting for 4 h.

#### Cell culture

C2C12 myocytes were cultured as previously described.^64^ Briefly, C2C12 myoblasts were grown at 37 °C in a 5% CO_2_ incubator in Dulbecco’s modified Eagle’s medium (DMEM) (4.5 g/L glucose) (Nacalai Tesque Corp., Kyoto, Japan) supplemented with 10% fetal bovine serum (Equitech-Bio, Inc., Kerrville, TX, USA) and 1% penicillin-streptomycin (Nacalai Tesque Corp.). To induce cell differentiation into myotubes, the medium was changed to DMEM (1.0 g/L glucose) (Nacalai Tesque Corp.) supplemented with 2% horse serum (Thermo Fisher Scientific Inc.) and 1% penicillin-streptomycin (Nacalai Tesque Corp.) for 4 days. Then, experiments using BAs (αMCA and tauro-αMCA; Cayman Chemical, Ann Arbor, MI, USA; Toronto Research Chemical, ON, Canada), palmitic acid (TCI Chemicals, Tokyo, Japan), and sotrastaurin, a PKCθ inhibitor (AEB071; Selleck Chemicals, Houston, TX, USA), were performed following incubation for 4 h in non-supplemented medium.

### Method details

#### GTTs and ITTs

In the recipient mice, oral GTT was performed after an overnight fast. The mice received 20% glucose solution (FUSO Pharmaceutical Industries, Osaka, Japan) by gavage at 100 μL/10 g body weight. Tail vein blood was collected immediately before and at 30, 60, and 120 min after gavage. Glucose levels were measured using Glutest Ace R (Sanwa Kagaku Kenkyusho Co. Ltd. Nagoya, Japan). For ITT, mice were starved for 4 h and 0.33 U/kg human insulin (Humulin R; Eli Lilly Japan K.K., Kobe, Japan) was injected intraperitoneally. Glucose levels were measured from tail vein blood samples collected immediately before and at 30, 60, and 120 min after insulin injection.

#### Analysis of fecal microbiota by high throughput 16S rRNA amplicon sequencing

Extraction of bacterial DNA from feces was conducted as described previously.^65^ Library preparation and deep sequencing were also performed as described previously.^65^ In the DNA sequencing, the V3-4 region of 16S rRNA genes in each sample was amplified by two-step PCR. The prepared library pool combined with phiX Control (expected 20%) was sequenced using a 285-bp paired-end strategy on the MiSeq (Illumina KK, Tokyo, Japan) according to the manufacturer’s instructions. Afterward, sequencing data analysis was performed with QIIME2 (version 2020.8) as described previously.^66^ In the process, the sequence was denoised with a DADA2 plugin of QIIME2. The Sklearn classifier was used for taxonomic assignment against the Greengenes database (13_8).

#### Plasma glucose and insulin

Plasma glucose levels were measured using a glucose CII test kit (Wako, Osaka, Japan). Plasma insulin levels were measured using an enzyme-linked immunosorbent assay (ELISA) kit as per the manufacturer’s instructions (Mercodia AB, Uppsala, Sweden).

#### Plasma metabolome analysis

Plasma samples from three recipient mice that received FMT for 1 week were used for targeted metabolomic analysis at Human Metabolome Technology Inc. (Tsuruoka, Yamagata, Japan). Capillary electrophoresis time-of-flight mass spectrometry (CE-TOF-MS) analysis to detect approximately 1,000 water-soluble and ionic metabolites including glucose phosphates, amino acids, peptides, nucleic acids, organic acids, vitamins, and fatty acids was performed using an Agilent CE capillary electrophoresis system equipped with an Agilent 6210 time-of-flight mass spectrometer (Agilent Technologies, Waldbronn, Germany). Briefly, plasma (50 µL) was added to methanol (200 µL) containing internal standards (H3304-1002, HMT). The solution was mixed with Milli-Q water (150 µL) and centrifugally filtered through a Millipore 5-kDa cutoff filter (Millipore, Bedford, MA, USA). The filtrate was then resuspended in Milli-Q water (50 µL) for CE-TOFMS analysis using an Agilent CE capillary electrophoresis system equipped with an Agilent 6210 time-of-flight mass spectrometer (Agilent Technologies, Inc., Santa Clara, CA, USA). The spectrometer was scanned from *m/z* 50 to 1,000, and peaks were extracted using the MasterHands automatic integration software (Keio University, Tsuruoka, Yamagata, Japan) to obtain peak information, including *m/z*, peak area, and migration time (MT). Signal peaks were annotated according to HMT’s metabolite database based on their *m*/*z* values and MTs. Areas of the annotated peaks were then normalized to internal standards and sample amount in order to obtain the relative levels of each metabolite.

#### Plasma BA analysis

Plasma BA profiles were detected using a liquid chromatography-tandem mass spectrometry (LC-MS/MS) system.^67^ Briefly, mouse plasma (20 µL) was diluted 100-fold with ^2^H-labeled internal standards and 0.5 M potassium phosphate buffer (pH 7.4). The mixture was applied to a Bond Elut C18 cartridge (200 mg; Agilent Technologies, Santa Clara, CA, USA). The target molecules were eluted in water/ethanol (1:9, v/v). The eluate was evaporated under nitrogen until dry and dissolved in 20 mM ammonium acetate buffer (pH 7.5)/methanol (1:1, v/v). An aliquot of each sample was injected into the LC-MS/MS system for analysis. Chromatographic separation was performed using a Hypersil GOLD column (Thermo Fisher Scientific, Waltham, MA). A mixture of 20 mM ammonium acetate buffer (pH 7.5), acetonitrile, and methanol (70:15:15, v/v) was used for the initial mobile phase, which was gradually changed to 30:35:35 (v/v/v) over 30 min.

#### Microarray analysis

Total RNA was extracted from the frozen gastrocnemius muscle of recipient mice using an RNeasy Mini Kit (Qiagen, Valencia, CA, USA). Target hybridization and cRNA preparations were performed according to the Affymetrix GeneChip® Technical Protocol (Affymetrix, Santa Clara, CA, USA). Affymetrix GeneChip® Mouse Gene 1.0 ST arrays were stained and washed in an Affymetrix Fluidics Station 450 and scanned using a GeneChip® Scanner 3000 7G (Affymetrix). Expression levels were analyzed using Transcriptome Analysis Console Software 4.0 (Affymetrix) following background correction, signal summarization, and normalization by SST[EMS1]-RMA. Pathways that were significantly enriched in the list of differentially expressed genes were identified using Ingenuity® Pathway Analysis (IPA®, QIAGEN).

#### Glucose uptake assay

2-deoxy-glucose uptake was examined using a Glucose Uptake-Glo Assay (Promega Corp., Madison, WI, USA). Differentiated myotubes were prepared in 96 well plates. After washing with PBS, cholic acids were added to the cells and cultured with palmitic acid for 24 h. Thereafter, reagents were added according to the manufacturer’s instructions. 2-deoxy-glucose uptake was measured as luminescence intensity using a SpectraMax microplate reader (Molecular Devices, LLC., Sunnyvale, CA, USA).

#### Glycogen assay

The gastrocnemius muscle was homogenized with 0.3 M percholic acid on ice. Then, 200 mM sodium acetate and 4.2 mg/mL amyloglucosidase were added and the sample was incubated for 2 h at 55°C. Thereafter, 2 M Tris-HCl was added at room temperature. After centrifugation, glucose content was examined using a D-glucose measurement kit as per the manufacturer’s instructions (F-kit glucose, Roche Diagnostics, Mannheim, Germany).

#### Protein assay

Extracted proteins were separated by SDS-PAGE and transferred onto nitrocellulose membranes. Subsequently, the blots were incubated with primary antibodies against phospho-AMPKα (Thr172), total AMPKα, phospho-ACC (Ser212), total ACC, phospho-Akt (Ser473), total Akt, phospho-Akt substrate (Ser/Thr) (AS), phospho-AS160 (Thr642), total AS160, phospho-calcium/calmodulin-dependent protein kinase II (CaMKII) (Thr286), total CaMKII, phospho-LKB1 (Ser428), total LKB1 (all from Cell Signaling Technology, Beverly, MA), glucose transporter 4 (GLUT4) (Merck Millipore, Darmstadt, Germany), and glyceraldehyde-3-phosphate dehydrogenase (GAPDH) (Abcam, Cambridge, MA, USA). Subsequently, membranes were incubated with horseradish peroxidase-conjugated secondary antibody and visualized using enhanced chemiluminescence substrate (Immobilon, Millipore). Each band was detected using an image analyzer (Lumino Graph I, ATTO Corp., Tokyo, Japan). Signal intensities were quantified using the ImageJ software (National Institutes of Health, Bethesda, MD). C-C motif chemokine ligand 2 (CCL-2) and IGF-1 in tissues and culture media were measured using ELISA kits (MCP-1; Sigma, IGF-1; Proteintech, Chicago, IL, USA).

#### RNA extraction and real-time PCR

Total RNA was extracted using Sepazol (Nacalai Tesque). After reverse transcription, quantitative PCR was performed using a StepOne Plus Real-Time PCR system (Life Technologies, Carlsbad, CA, USA) with the THUNDERBIRD Probe qPCR Mix (ToYoBo, Tokyo, Japan) and TaqMan primers *CCL-2*: ID Mm00441242_m1, C-X-C motif ligand 1 [*CXCL-1*]: ID Mm04207460_m1, toll-like receptor 4 [*TLR-4*]: ID Mm00445273_m1, tumor necrosis factor-α [*TNF-α*]: ID Mm00443258_m1, interleukin-1β [*IL-1β*]: ID Mm00434228_m1, *F4/80*: ID Mm00802529_m1, cytochrome c oxidase subunit IV isoform 1 [*COX4-1*]: ID Mm01250094_m1, and peroxisome proliferator-activated receptor gamma coactivator 1-α [*PGC1α*]: ID Ms01208835_m1, Thermo Fisher Scientific). Threshold cycle (Ct) values were determined using the StepOne software, version 2.3 (Thermo Fisher Scientific). Relative gene expression was calculated by the comparative Ct method relative to the *β-actin* reference gene.

#### Fecal bile salt hydrolase activity

Total fecal protein sample was extracted with pre-deoxygenated phosphate buffer added with bacterial and mammalian protease inhibitors (Sigma) and 1 mM dithiothreitol. The bile salt hydrolase activity was measured using a modification a precipitation-based assay described previously.^68^^,^^69^ The extracted samples (500 μg protein) were incubated with 1 mM tauro-αMCA in PBS (pH 5.8) at 37 °C for 6 h. The insoluble free-form product was measured as absorbance intensity using a microplate reader (Molecular Devices). The protein samples incubated with PBS or αMCA were set as negative and positive controls, respectively.

#### Network analysis

To identify possible networks between microbiota, the network analysis of plasma BA deconjugation, and muscle AMPK signaling was analyzed with Spearman’s correlations, and visualized with the Cytoscape v3.8.2 open-source software. Bacteria that showed higher abundance in RT than RS at 1 week of FMT were used for the analysis. Spearman correlation coefficients with a minimal cutoff threshold of 0.6 (p < 0.05, false discovery rate corrected) were calculated.

### Quantification and statistical analysis

All data are reported as the mean ± standard error. ANOVA or Student’s t-test was performed to determine statistical significance between groups. If ANOVA indicated statistical significance, *post hoc* multiple comparisons were conducted using Tukey’s honestly significant difference test to determine the significance of the differences among the mean values. Microarray data were normalized using the robust multi-array average method (Expression Console 1.3.0.187, Affymetrix) and analyzed using one-way between-subject ANOVA (recipient from sedentary donor [RS] vs. recipient from trained donor [RT]). Screening of significant changes in expressed transcripts was considered at p < 0.05 and false discovery rate (FDR) < 0.1. Correlations between microbiota and BAs and between mRNA and BAs were evaluated using Spearman’s correlation analysis. When a normal distribution was not obtained, a non-parametric analysis was used for each comparison. Statistical significance was set at p < 0.05, a trend at p < 0.1.

## Data Availability

Complete data of the microarray analysis have been deposited at GEO and are publicly available as of the date of publication. Accession numbers are listed in the key resources table. This paper does not report original code. Any additional information required to reanalyze the data reported in this paper is available from the lead contact upon request.
