# Supplementary material for: Exercise-acclimated microbiota improves skeletal muscle metabolism via circulating bile acid deconjugation
Source: iScience. 2023 Feb 21;26(3):106251. doi: 10.1016/j.isci.2023.106251 (PMC10005909; doi:10.1016/j.isci.2023.106251)
Supplement: Document S1. Figures S1–S8 and Tables S1–S4 [file mmc1.pdf]

iScience, Volume *26*

Supplemental information

Exercise-acclimated microbiota improves skeletal muscle metabolism via circulating bile acid deconjugation

Wataru Aoi, Ryo Inoue, Katsura Mizushima, Akira Honda, Marie Björnholm, Tomohisa Takagi, and Yuji Naito

Table S1. Difference in gut microbiota between sedentary and trained donors, Related to Figure 1.

Change vs DS	Genus	Abundant (%)		*P*-value	Frequency (Present/Total (%))	
		DS	DT		DS	DT
Increase	*Lactococcus*	1.59 ± 0.59	3.56 ± 1.11	0.089	7/7 (100)	8/8 (100)
	Lactobacillus	0.97 ± 0.47	4.45 ± 1.43	0.031	6/7 (88)	6/8 (86)
	Staphylococcus	0.00 ± 0.00	0.02 ± 0.01	0.033	2/7 (29)	5/8 (58)
Decrease	*Parabacteroides*	2.47 ± 0.63	1.17 ± 0.24	0.066	7/7 (100)	8/8 (100)
	Ruminococcus	2.46 ± 0.35	1.46 ± 0.40	0.057	7/7 (100)	8/8 (100)
	Roseburia	0.40 ± 0.11	0.17 ± 0.11	0.095	7/7 (100)	2/8 (25)
	Unclassified f_*Christensenellaceae*	0.19 ± 0.03	0.10 ± 0.02	0.030	7/7 (100)	8/8 (100)
	Unclassified f_*Erysipelotrichaceae*	0.06 ± 0.01	0.01 ± 0.00	0.008	7/7 (100)	3/8 (38)
	Pseudobutyrivibrio	0.04 ± 0.02	0.00 ± 0.00	0.087	2/7 (29)	0/8 (0)
	Unclassified c_*Clostridia*	0.02 ± 0.01	0.00 ± 0.00	0.040	4/7 (57)	1/8 (13)

The relative abundance of microbiota genera that altered ($p < 0.1$) between sedentary and trained donors. DS; sedentary donor, DT; trained donor. The results of abundant are presented as the mean ± standard error, with n = 7-8.

Table S2. Differences in gut microbiota between recipient mice at 1 w after fecal microbiota transplantation, Related to Figure 1.

Change vs RS	Genus	Abundant (%)		*P*-value	Frequency (Present/Total (%))	
		RS	RT		RS	RT
Increase	*Lactococcus*	2.16 ± 0.91	4.21 ± 0.95	0.092	5/6 (83)	5/6 (83)
	f_*Lachnospiraceae* g_*[Ruminococcus]*	0.70 ± 0.33	2.83 ± 0.79	0.029	4/6 (67)	6/6 (100)
	Lactobacillus	0.47 ± 0.22	10.05 ± 5.30	0.080	6/6 (100)	5/6 (83)
	Turicibacter	0.04 ± 0.03	0.34 ± 0.07	0.055	3/6 (50)	5/6 (83)
	Desulfovibrio	0.08 ± 0.03	0.22 ± 0.07	0.055	4/6 (67)	5/6 (83)
	Unclassified c_*Clostridia*	0.01 ± 0.01	0.03 ± 0.01	0.092	3/6 (50)	5/6 (83)
	Staphylococcus	0.00 ± 0.00	0.15 ± 0.04	0.013	0/6 (0)	5/6 (83)
Decrease	Unclassified f_*Ruminococcaceae*	6.73 ± 0.47	3.85 ± 0.75	0.009	6/6 (100)	6/6 (100)
	Unclassified o_*Bacteroidales*	3.52 ± 1.10	0.85 ± 0.41	0.041	6/6 (100)	5/6 (83)
	Parabacteroides	2.48 ±0.58	0.73 ± 0.21	0.020	6/6 (100)	6/6 (100)
	Unclassified f_*RFP12*	1.83 ± 0.77	0.39 ± 0.36	0.084	3/6 (50)	1/6 (17)
	Phascolarctobacterium	0.88 ± 0.36	0.16 ± 0.14	0.069	3/6 (50)	1/6 (17)
	Prevotella	0.85 ± 0.36	0.15 ± 0.14	0.073	3/6 (50)	1/6 (17)
	CF231	0.74 ± 0.34	0.13 ± 0.12	0.084	3/6 (50)	1/6 (17)
	Treponema	0.46 ± 0.19	0.06 ± 0.06	0.062	3/6 (50)	1/6 (17)
	YRC22	0.44 ± 0.19	0.11 ± 0.10	0.096	3/6 (50)	1/6 (17)
	Clostridium2	0.37 ± 0.16	0.07 ± 0.06	0.073	3/6 (50)	1/6 (17)
	Unclassified f_*Pirellulaceae*	0.32 ± 0.13	0.07 ± 0.06	0.080	3/6 (50)	1/6 (17)
	Unclassified f_*Synergistaceae*	0.26 ± 0.11	0.04 ± 0.04	0.071	3/6 (50)	1/6 (17)
	Unclassified f_*Victivallaceae*	0.26 ± 0.11	0.05 ± 0.04	0.071	3/6 (50)	1/6 (17)
	p-75-a5	0.23 ± 0.11	0.02 ± 0.02	0.073	3/6 (50)	1/6 (17)
	Unclassified f_*Christensenellaceae*	0.22 ± 0.08	0.08 ± 0.02	0.084	3/6 (50)	6/6 (100)
	Unclassified f_*F16*	0.20 ± 0.09	0.04 ± 0.04	0.093	3/6 (50)	1/6 (17)
	Fusobacterium	0.18 ± 0.08	0.03 ± 0.03	0.079	3/6 (50)	1/6 (17)
	Epulopiscium	0.18 ± 0.08	0.03 ± 0.03	0.072	3/6 (50)	1/6 (17)
	Unclassified f_*[Cerasicoccaceae]*	0.18 ± 0.08	0.04 ± 0.03	0.098	3/6 (50)	1/6 (17)

Table S2. (*Continued*)

Change vs RS	Genus	Abundant (%)		*P*-value	Frequency (Present/Total (%))	
		RS	RT		RS	RT
Decrease	*BF311*	0.15 ± 0.07	0.03 ± 0.03	0.097	3/6 (50)	1/6 (17)
	Fibrobacter	0.14 ± 0.06	0.03 ± 0.03	0.096	3/6 (50)	1/6 (17)
	Unclassified f_*[Tissierellaceae]*	0.12 ± 0.05	0.01 ± 0.01	0.054	3/6 (50)	1/6 (17)
	Unclassified f_*Coriobacteriaceae*	0.11 ± 0.05	0.00 ± 0.01	0.069	3/6 (50)	1/6 (17)
	Unclassified o_*YS2*	0.10 ± 0.05	0.02 ± 0.01	0.079	3/6 (50)	1/6 (17)
	Anaerofilum	0.08 ± 0.03	0.01 ± 0.01	0.064	3/6 (50)	1/6 (17)
	Unclassified c_*Opitutae*	0.07 ± 0.04	0.01 ± 0.01	0.095	3/6 (50)	1/6 (17)
	Lachnobacterium	0.07 ± 0.03	0.01 ± 0.01	0.073	3/6 (50)	1/6 (17)
	Citrobacter	0.07 ± 0.03	0.01 ± 0.01	0.081	6/6 (100)	1/6 (17)
	Pseudoramibacter_Eubacterium	0.05 ± 0.02	0.01 ± 0.01	0.079	3/6 (50)	1/6 (17)
	Sedimentibacter	0.05 ± 0.02	0.01 ± 0.01	0.063	2/6 (33)	1/6 (17)
	Pseudobutyrivibrio	0.05 ± 0.03	0.00 ± 0.00	0.095	2/6 (33)	0/6 (0)
	Methanobrevibacter	0.05 ± 0.02	0.00 ± 0.00	0.056	2/6 (33)	1/6 (17)
	Anaerovibrio	0.03 ± 0.01	0.00 ± 0.00	0.065	2/6 (33)	1/6 (17)
	Morganella	0.03 ± 0.01	0.00 ± 0.00	0.054	2/6 (33)	0/6 (0)
	Unclassified o_*PL-11B10*	0.02 ± 0.01	0.00 ± 0.00	0.069	2/6 (33)	1/6 (17)
	RFN20	0.02 ± 0.01	0.00 ± 0.00	0.093	2/6 (33)	0/6 (0)
	Unclassified f_*R4-45B*	0.01 ± 0.01	0.00 ± 0.00	0.088	3/6 (50)	0/6 (0)
	Unclassified p_*Proteobacteria*	0.01 ± 0.01	0.00 ± 0.00	0.090	2/6 (33)	0/6 (0)
	Unclassified o_*RF32*	0.01 ± 0.00	0.00 ± 0.00	0.043	2/6 (33)	0/6 (0)
	Unclassified f_*Dehalobacteriaceae*	0.01 ± 0.01	0.00 ± 0.00	0.098	3/6 (50)	0/6 (0)
	Unclassified f_*Pseudoalteromonadaceae*	0.01 ± 0.01	0.00 ± 0.00	0.088	3/6 (50)	0/6 (0)
	Anaerostipes	0.01 ± 0.00	0.00 ± 0.00	0.087	3/6 (50)	0/6 (0)
	Caloramator	0.01 ± 0.00	0.00 ± 0.00	0.087	3/6 (50)	0/6 (0)
	Unclassified o_*PeHg47*	0.01 ± 0.00	0.00 ± 0.00	0.089	2/6 (33)	0/6 (0)
	Unclassified f_*RF16*	0.01 ± 0.00	0.00 ± 0.00	0.089	3/6 (50)	0/6 (0)

The relative abundance of microbiota genera that altered ($p < 0.1$) between sedentary and trained recipients at 1 week after FMT. RS; recipient from sedentary donor, RT; recipient from trained donor. The results of abundant are presented as the mean ± standard error, with $n = 6$.

Table S3. Differences in gut microbiota between recipient mice at 8 w after fecal microbiota transplantation, Related to Figure 1.

Change vs RS	Genus	Abundant (%)		*P*-value	Frequency (Present/Total (%))	
		RS	RT		RS	RT
Increase	Unclassified f_*Lachnospiraceae*	6.10 ± 1.33	9.48 ± 1.34	0.059	8/8 (100)	8/8 (100)
	Unclassified f_*Desulfovibrionaceae*	3.45 ± 0.99	6.58 ± 1.38	0.037	8/8 (100)	8/8 (100)
	f_*Lachnospiraceae* g_*[Ruminococcus]*	2.56 ± 0.39	6.38 ± 1.18	0.010	8/8 (100)	8/8 (100)
	Ruminococcus	0.79 ± 0.20	1.50 ± 0.17	0.012	8/8 (100)	8/8 (100)
	Mucispirillum	0.48 ± 0.19	3.05 ± 0.91	0.017	8/8 (100)	8/8 (100)
	Coprococcus	0.23 ± 0.06	0.42 ± 0.06	0.029	4/8 (50)	8/8 (100)
	Desulfovibrio	0.18 ± 0.03	0.41 ± 0.07	0.011	8/8 (100)	8/8 (100)
	Roseburia	0.17 ± 0.10	0.85 ± 0.34	0.054	8/8 (100)	6/8 (75)
	Bilophila	0.03 ± 0.01	0.09 ± 0.03	0.042	6/8 (75)	7/8 (88)
	Unclassified f_*Erysipelotrichaceae*	0.02 ± 0.01	4.06 ± 0.02	0.031	4/8 (50)	6/8 (75)
	Unclassified c_*Clostridia*	0.00 ± 0.00	0.00 ± 0.00	0.066	1/8 (13)	4/8 (50)
Decrease	Unclassified f_*S24-7*	15.36 ± 1.59	11.87 ± 1.58	0.084	8/8 (100)	8/8 (100)
	Allobaculum	11.21 ± 4.17	4.68 ± 1.34	0.099	8/8 (100)	7/8 (88)
	Bifidobacterium	1.54 ± 0.70	0.35 ± 0.13	0.081	8/8 (100)	5/8 (63)
	Unclassified f_*Enterobacteriaceae*	0.46 ± 0.19	0.05 ± 0.02	0.048	6/8 (75)	4/8 (50)
	Clostridium	0.29 ± 0.09	0.07 ± 0.05	0.040	8/8 (100)	4/8 (50)
	Blautia	0.15 ± 0.09	0.01 ± 0.01	0.099	6/8 (75)	1/8 (13)
	Unclassified f_*Aerococcaceae*	0.01 ± 0.01	0.00 ± 0.00	0.099	3/8 (38)	0/8 (0)

The relative abundance of microbiota genera that altered ($p < 0.1$) between sedentary and trained recipients at 8 week after FMT. RS; recipient from sedentary donor, RT; recipient from trained donor. The results of abundant are presented as the mean ± standard error, with $n = 8$.

Table S4. Plasma metabolome profile of recipient mice, Related to Figure 2.

Compound	*m/z*	MT/RT	RS	RT	RT vs RS (Ratio)
Cholic acid	407.3	6.9	1.3E-04	3.6E-04	2.82
1-Methylnicotinamide	137.1	6.8	8.0E-05	1.9E-04	2.39
3-Indoxylsulfuric acid	212.0	9.7	3.8E-04	8.5E-04	2.21
Phosphocreatine	210.0	12.4	2.3E-04	4.8E-04	2.10
Adenosine	268.1	9.0	2.4E-05	4.4E-05	1.89
1*H*-Imidazole-4-propionic acid	141.1	7.4	8.3E-05	1.5E-04	1.87
4-Methyl-2-oxovaleric acid<br>3-Methyl-2-oxovaleric acid<br>2-Oxohexanoic acid	129.1	9.7	2.3E-03	4.1E-03	1.79
N-(*o*-Toluoyl)glycine<br>Phenaceturic acid	192.1	8.3	2.8E-04	5.1E-04	1.79
Isobutyrylcarnitine	232.2	8.7	2.5E-04	4.3E-04	1.76
S-Sulfocysteine	200.0	11.3	5.0E-04	8.7E-04	1.74
4-Oxopyrrolidine-2-carboxylic acid	130.0	9.9	1.3E-04	2.3E-04	1.69
Trimethylamine *N*-oxide	76.1	6.1	3.7E-04	6.2E-04	1.67
Glu	148.1	9.9	1.1E-02	1.8E-02	1.59
Homocarnosine	241.1	6.3	2.0E-05	3.1E-05	1.58
Butyrylcarnitine	232.2	8.7	9.5E-04	1.5E-03	1.57
Asp	134.0	10.4	1.7E-03	2.7E-03	1.55
Guanidoacetic acid	118.1	7.6	5.6E-04	8.5E-04	1.54
2-Oxoglutaric acid	145.0	21.8	3.9E-03	6.1E-03	1.54
N^1-Methyl-4-pyridone-5-carboxamide	153.1	16.2	2.0E-04	3.0E-04	1.53
Pyridoxal	168.1	8.0	7.0E-05	1.1E-04	1.52
Galactosamine<br>Glucosamine	180.1	8.5	1.2E-04	1.8E-04	1.51
1-Methyl-4-imidazoleacetic acid	141.1	7.6	6.8E-05	1.0E-04	1.50

The metabolites with 1.5-fold higher in recipients from trained donors than sedentary donors. RS; recipient from sedentary donor, RT; recipient from trained donor. Results are presented as the mean ± standard error, with $n = 3$.

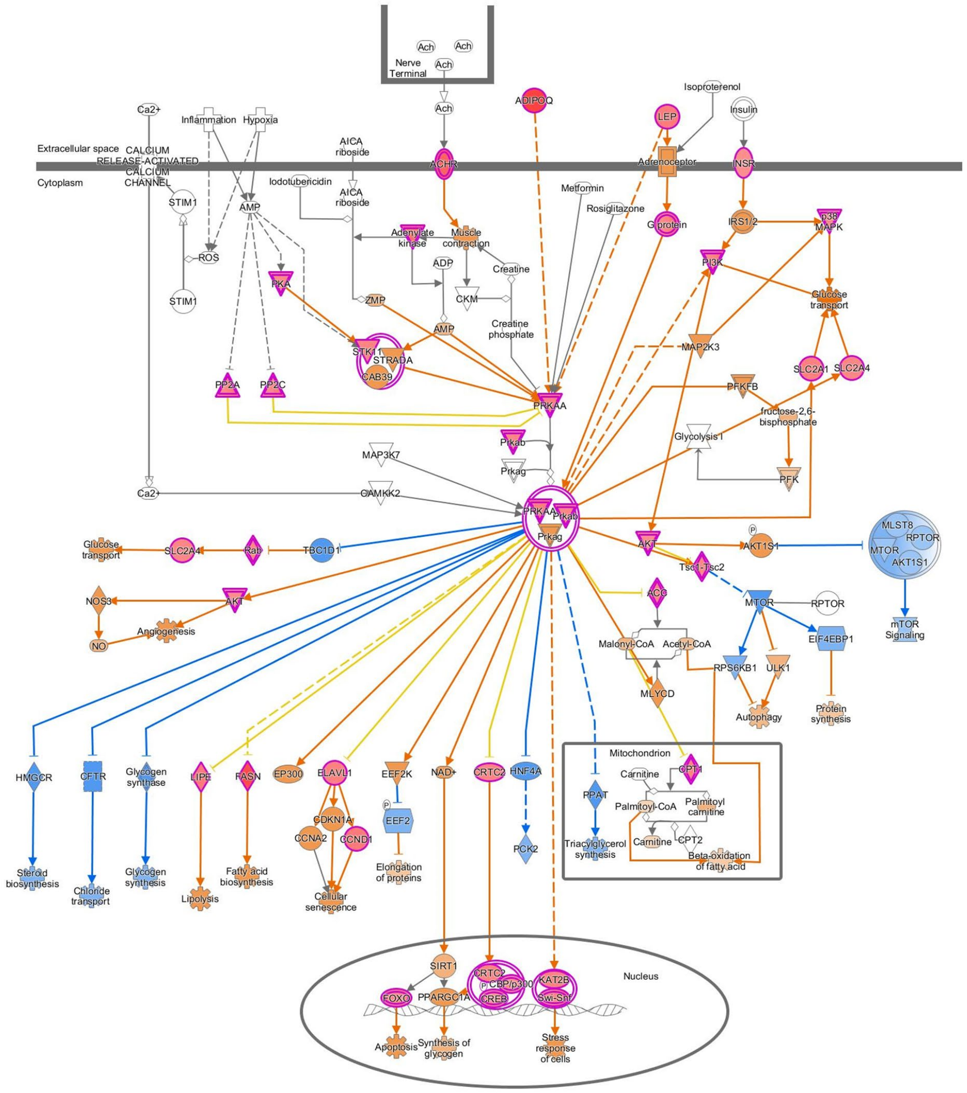

Figure S1. Upregulation of AMPK signaling factors in skeletal muscle from recipient mice, Related to Figure 3. Ingenuity canonical pathway analysis using transcriptome results revealed that the AMP-activated kinase (AMPK) signaling was highly ranked in pathways activated by fecal microbiota transplantation from trained donors. The factors in red (validated in dataset, $p < 0.05$ and false discovery rate < 0.1) and orange (predicted) showed upregulation in the RT mice than RS mice. The factors in blue showed predicted downregulation in the RT mice than RS mice. Color intensity shows the degree of regulation. The orange lines between factors show “leads to activation” and the blue lines between factors show “leads to inhibition”. The factors in vertical circle, transmembrane receptor; horizontal circle, transcriptional regulator; circle, complex/group/other; triangle, phosphatase; reverse triangle, kinase; diamond, enzyme.

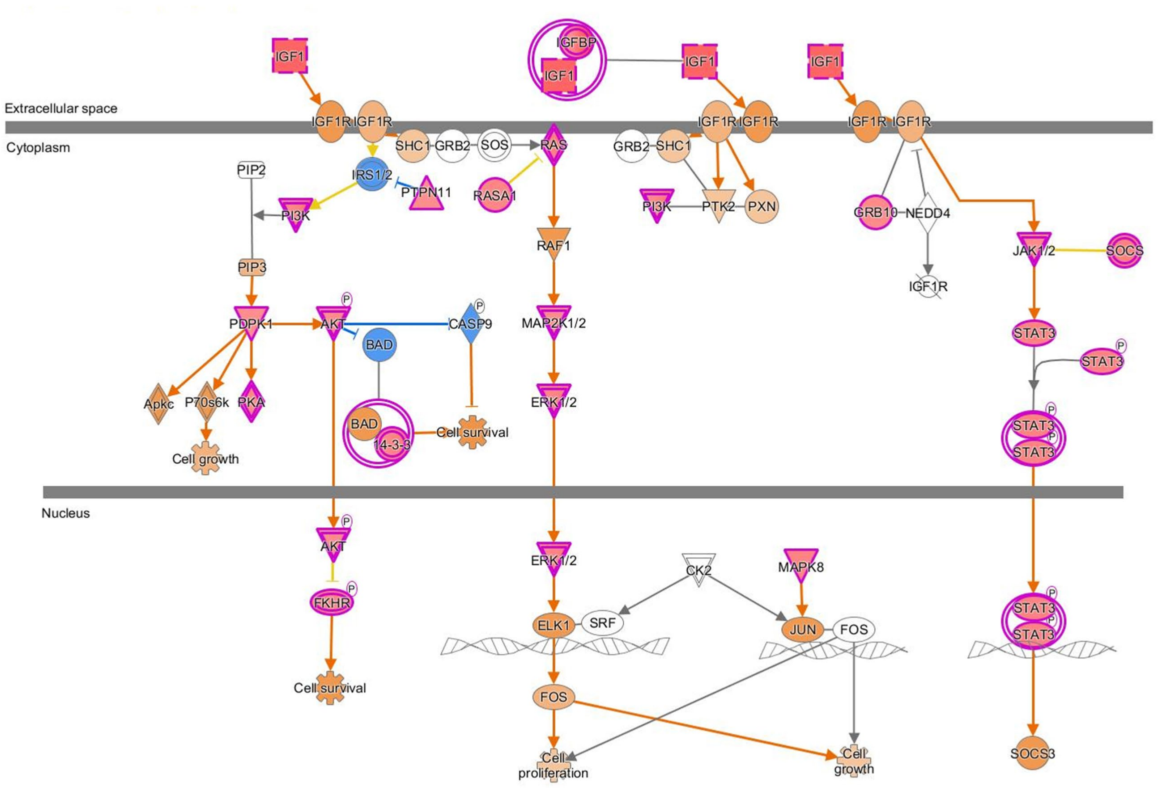

Figure S2. Upregulation of IGF-1 signaling factors in skeletal muscle from recipient mice, Related to Figure 3. Ingenuity canonical pathway analysis using transcriptome results revealed that the insulin growth factor (IGF-1) signaling was highly ranked in pathways activated by fecal microbiota transplantation from trained donors. The factors in red showed higher values in the RT mice than RS mice ($p < 0.05$ and false discovery rate < 0.1). The factors in blue show predicted downregulation in the RT mice than RS mice. Color intensity of them shows the degree of regulation. The orange lines between factors show "leads to activation" and the blue lines between factors showed "leads to inhibition". The factors in vertical circle, transmembrane receptor; horizontal circle, transcriptional regulator; circle, complex/group/other; triangle, phosphatase; reverse triangle, kinase; diamond, enzyme.

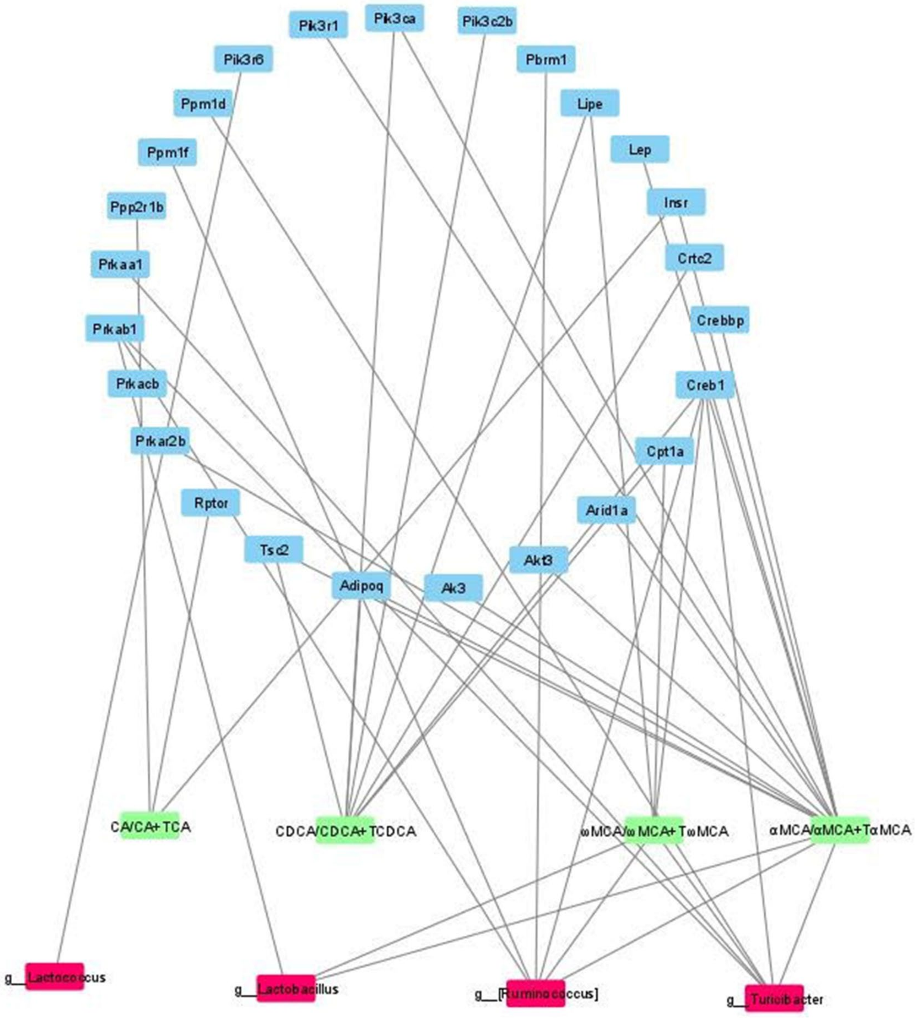

Figure S3. Correlations between AMPK signaling, plasma bile acids (BAs), and intestinal microbiota, Related to Figure 3. A network analysis of the transcriptome, plasma BAs, and the abundance of bacterial genera revealed that *Lactobacillus*, *Lactococcus*, [*Ruminococcus*], and *Turicibacter* affected AMPK signaling factors in the BA-dependent or independent routes. Spearman correlation coefficients with a minimal cutoff threshold of 0.6 ($p < 0.05$, false discovery rate corrected) were calculated. Factors with blue represent AMPK signaling genes. Factors in green represent deconjugated ratio of bile acids, CA; choric acid, CDCA; chenodeoxycholic acid, MCA; murichoric acid. Factors in red indicate microbiota genera.

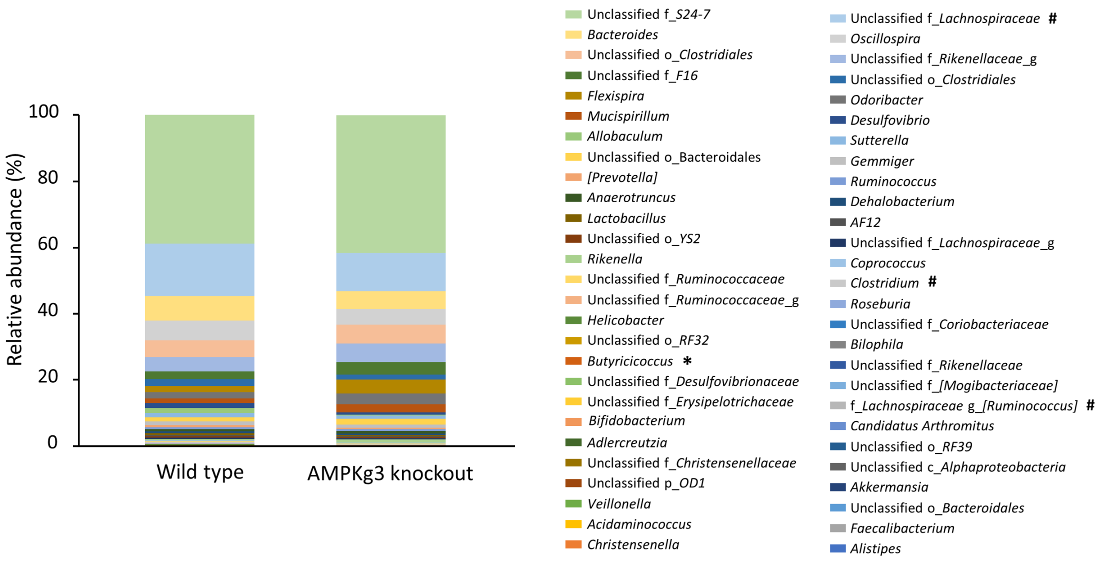

Figure S4. Microbiota profile in wild-type and AMPKγ3 knockout mice, Related to Figure 3. Difference of genera abundance profile in wild-type and AMPKγ3 knockout mice (n = 3). $^{\#}p < 0.1$ and $^{*}p < 0.05$, genera decreased in AMPKγ3 knockout mice compared to that in wild-type mice.

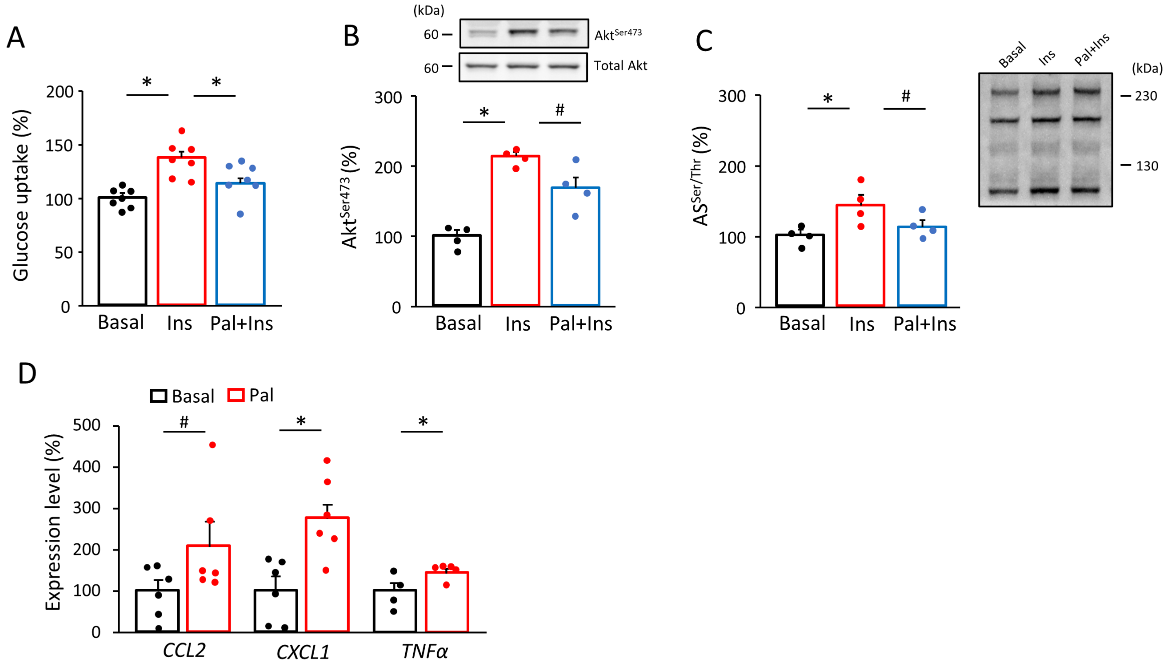

Figure S5. The responses of insulin signaling and inflammatory factors to palmitic acid in cultured myotubes, Related to Figure 4. Glucose uptake (A), Akt^{Ser473} (B) and $AS^{Ser/Thr}$ (C) phosphorylation with insulin (25 nM) for 15 min following with or without palmitic acid (200 μM) for 24 h in C2C12 myotubes. mRNA levels of *CCL-2*, *CXCL-1*, and *TNF-α* (D) with or without palmitic acid for 24 h in myotubes. Ins; insulin, Pal; palmitic acid. $^{\#}p < 0.1$ and $^{*}p < 0.05$ between conditions. Results are presented as the mean ± standard error, with $n = 4$-7 per group.

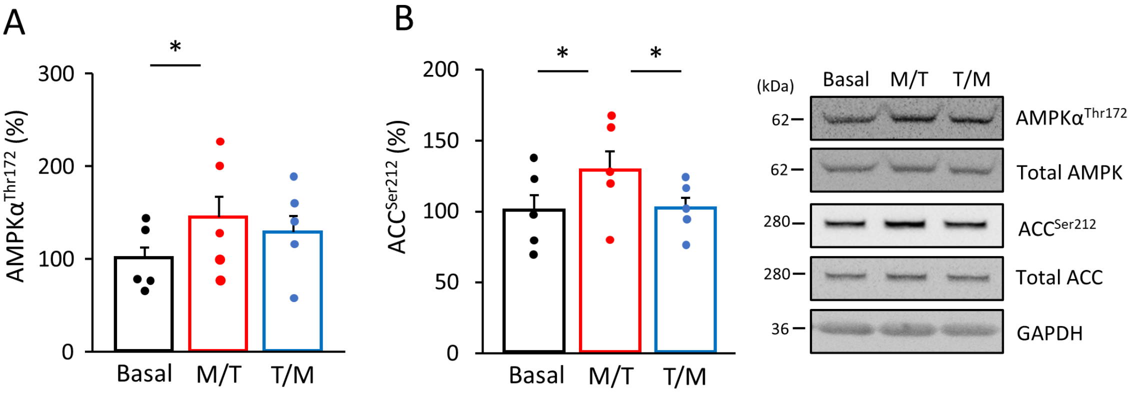

Figure S6. Ratios of the different forms of BA modulate metabolic responses in cultured myotubes, Related to Figure 4. AMPKα^{Thr172} (A) and ACC^{Ser212} (B) phosphorylation with or without palmitic acid (200 µM) in the high MCA and low TMCA (6.7 and 3.3 [uM]) or low MCA and high TMCA (3.3 and 6.7 [uM]) for 24 h in C2C12 myotubes. M/T; high MCA and low TMCA (6.7 and 3.3 [uM]), T/M; low MCA and high TMCA (3.3 and 6.7 [uM]). $*p < 0.05$ between conditions. Results are presented as the mean ± standard error, with $n = 5$ per group.

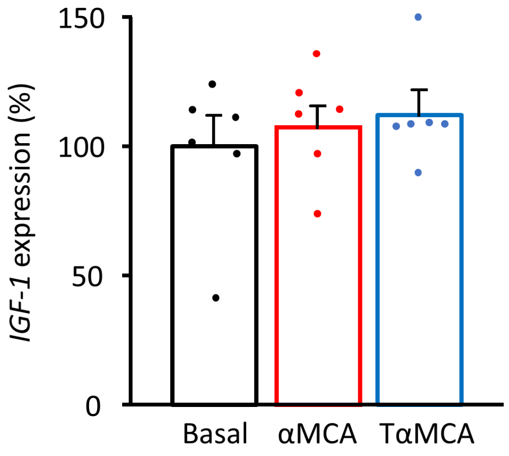

Figure S7. Effect of free-form and tauro-conjugated BAs on IGF-1 response in cultured myotubes, Related to Figure 4. *IGF-1* mRNA levels in the absence or presence of αMCA and TαMCA. Results are presented as the mean ± standard error, with $n = 6$.

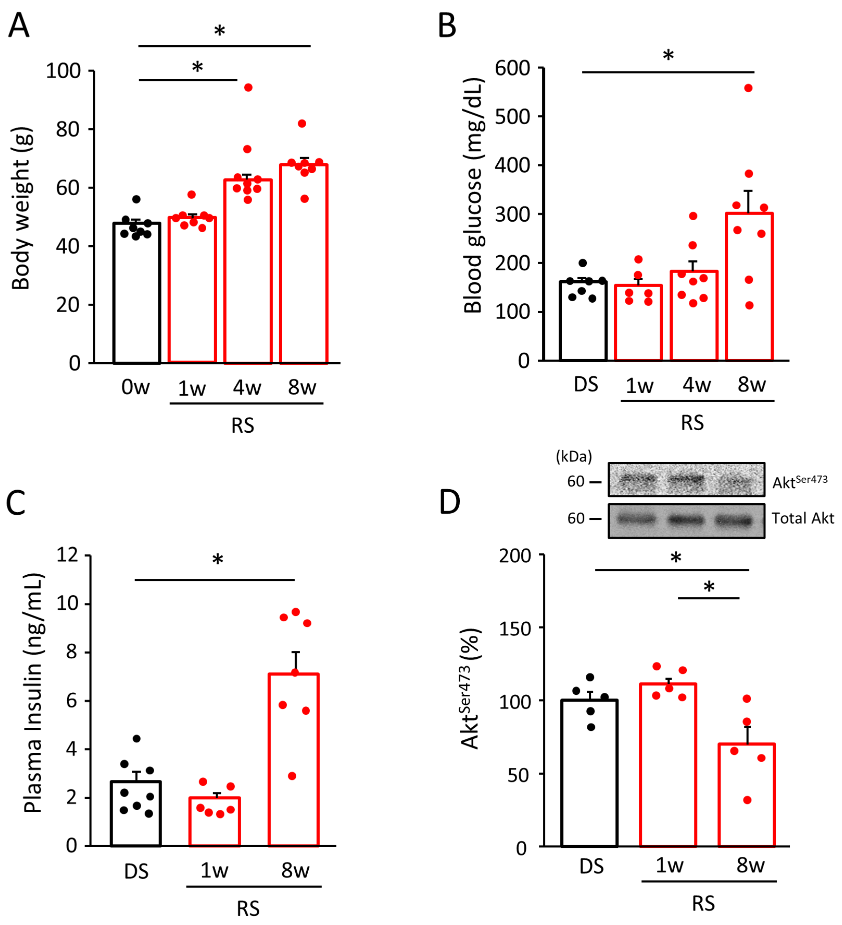

Figure S8. Time-course changes of metabolic parameters in HFD-fed mice, Related to Figure 5. Body weight (A), blood glucose (B), and plasma insulin (C) in recipient mice fed with HFD. Akt^{Ser473} phosphorylation in gastrocnemius muscle (D) in RS mice fed with HFD or DS mice fed with normal chow. RS; recipient from sedentary donor, RT; recipient from trained donor. *$p < 0.05$ between groups. Results are presented as the mean ± standard error, with n = 5-8 per group.
